# Theoretical and molecular mechanistic investigations of novel (3-(furan-2-yl)pyrazol-4-yl) chalcones against lung carcinoma cell line (A549)

**DOI:** 10.1007/s00210-022-02344-x

**Published:** 2022-12-05

**Authors:** Magda F. Mohamed, Nada S. Ibrahim, Amna A. Saddiq, Omar A. Almaghrabi, Maha E. Al-Hazemi, Hamdi M. Hassaneen, Ismail A. Abdelhamid

**Affiliations:** 1 Department of Chemistry (Biochemistry Branch), Cairo University, Giza, 12613 Egypt; 2 Department of Biology, College of Science and Art at Khulis, University of Jeddah, Jeddah, Saudi Arabia; 3 Department of Biology, College of Science, University of Jeddah, Jeddah, Saudi Arabia; 4 Department of Chemistry, College of Science and Art at Khulis, University of Jeddah, Jeddah, Saudi Arabia; 5 Department of Chemistry, Cairo University, Giza, 12613 Egypt

**Keywords:** (3-(Furan-2-yl)pyrazol-4-yl)chalcone, MTT assay, A549, Docking studies, Two mechanism of apoptosis

## Abstract

A new chalcone series has been developed that may be useful in the treatment of lung cancer. The new series was confirmed by the different spectral tools. MTT assay was used to detect the cytotoxic effect of the novel chalcones against lung cancer cell line (A549). Molecular docking studies were performed on the most two effective chalcones **7b** and **7c**. Different molecular techniques were utilized to study the activity and the effect of two chalcones **7b** and **7c** on apoptosis of A549 cell line.

**Supplementary Information:**

The online version contains supplementary material available at 10.1007/s00210-022-02344-x.

## Introduction

One of the most common cancers that occurred in the world was lung cancer. Smoking is approximately the main risk factor for lung cancer. There were two types of lung carcinoma, the detection of them was performed in literature (Tantraworasin et al. 2013). Undesired effects and risks of the disease can be overcome if the disease was early detected and diagnosed. If the disease was not cured early, it may invade and spread rapidly to the neighbouring tissue and other organs of the body. Chalcones, exist in a conjugated form, where the two rings (A and B) are linked by the keto-ethylenic system (Lemes et al. 2020). The biological action of these compounds is thought to be due to the conjugation of the double bond with the carbonyl group. Chalcones are a subject of ongoing research since they exhibit a wide range of biological activities including antibacterial (Asiri and Khan 2011; Mohamed et al. 2012), antimalarial (Li et al. 1995), antioxidant (Bandgar et al. 2009; Shenvi et al. 2013), antiviral (Onyilagha et al. 1997), anti-inflammatory (Hsieh et al. 2000; Bekhit and Abdel-Aziem 2004), analgesic (Heidari et al. 2009) antiplatelet (Lin et al. 2001), and anti-cancer agents (Sashidhara et al. 2010; Shenvi et al. 2013). Pyrazoles are nitrogen-containing heterocycles that play an important role in medicinal chemistry due to their wide range of biological applications, including anticancer (Sangani et al. 2014; Metwally et al. 2015; Alam et al. 2016), anti-inflammatory (Farghaly et al. 2000; Kendre et al. 2015), anticonvulsant (Kaushik et al. 2010), antioxidant (Viveka et al. 2015; Bellam et al. 2017; Sallam et al. 2020), and antimicrobial activities (Viveka et al. 2015; Kendre et al. 2015). In addition, 3-(furan-2-yl)-1*H*-pyrazoles (Andicsová et al. 2018; Ryan et al. 2020; Barus et al. 2021) have been reported to have biologically interesting applications. Based on these findings, and in continuation of our study interest in bioactive heterocycle production (Mohamed et al. 2017, 2018, 2020, 2021a, b, c, Sroor et al. 2019, 2020; Fathi et al. 2021; Helmy et al. 2021; Ragheb et al. 2022; Salem et al. 2022; Waly Eldeen et al. 2022), we were inspired to synthesize the pyrazolyl-chalcones and evaluate their in vitro anti-cancer effectiveness against different human cancer cell lines with an emphasis on the novel two chalcones **7b**, and **7c** that proved strong and interesting cytotoxic effect against lung carcinoma (A549) cell line. To find their effect on the apoptotic process of cancer cells, many theoretical and experimental investigations were extensively performed.

## Results and discussions

### Chemistry

**Scheme 1:**
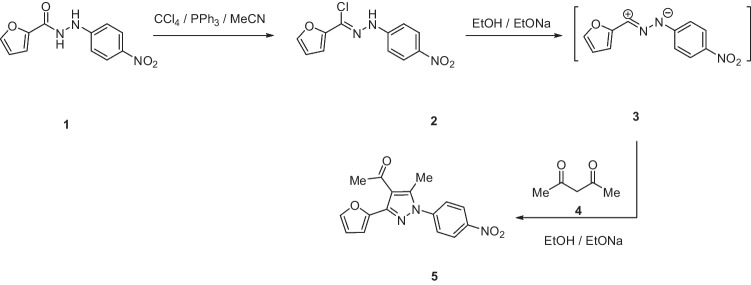
Synthesis of 1-(3-(furan-2-yl)-5-methyl-1-(4-nitrophenyl)-1*H*-pyrazol-4-yl)ethan-1-one **5**

The first step involves the chlorination of the respective *N*′-(4-nitrophenyl)furan-2-carbohydrazide **1** that affords *N*-(4-nitrophenyl)furan-2-carbohydrazonoyl chloride **2**. In the presence of ethanolic sodium ethoxide solution, compound **2** reacts with acetylacetone **4** to provide **5**. It is assumed that *N*-(4-nitrophenyl)furan-2-carbohydrazonoyl chloride **2** is converted into nitrilimine **3** that reacts with acetylacetone 4 through [3 + 2] cycloaddition that yields the final isolable products **5** (Scheme 1) (Hassaneen et al. 1988, 1991).

**Scheme 2:**
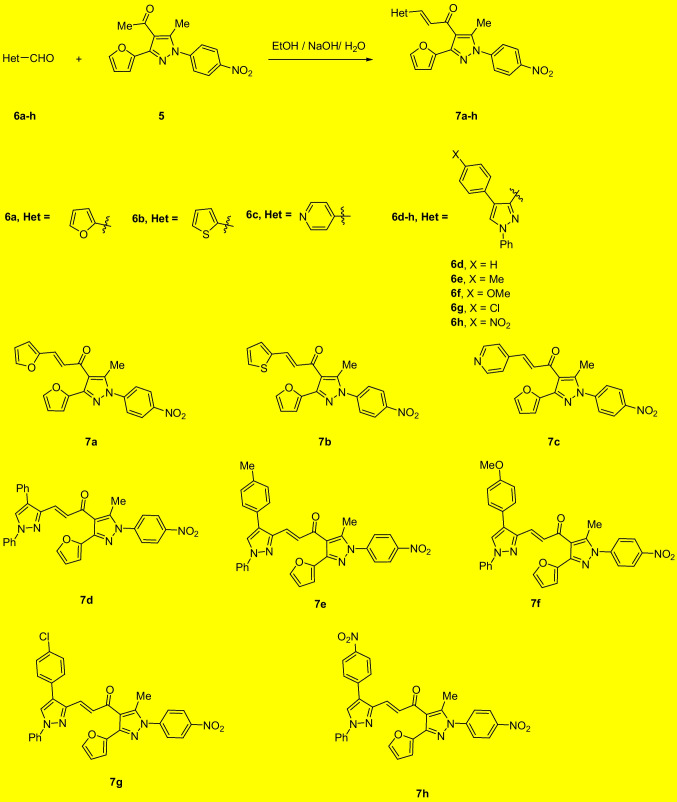
Synthesis of (3-(Furan-2-yl)pyrazol-4-yl) chalcones **7a-h**

The pyrazolyl-chalcones **7a-h** were produced by Claisen–Schmidt condensation of acetylpyrazole **5** with equimolar quantities of heteroarylaldehydes **6a-f** in ethanol in the presence of sodium hydroxide solution (Scheme 2). Based on spectral data analysis, the formed products’ structures were elucidated. As an example, ^1^H NMR spectrum of chalcone **7a** revealed one singlet signal at 2.60 for one methyl group. Also, ^1^H NMR of compound **7a** displayed two doublets of vinyl protons at δ 6.86 and 7.47 with coupling constant *J* = 16.2 Hz (which confirms the trans configuration of the two vinyl protons). Besides, the structure of **7a** was verified based on ^13^C NMR that indicated 19 signals corresponding to 19 different carbon atoms.

### Biological part

#### Structural activity relationship

**Scheme 3:**
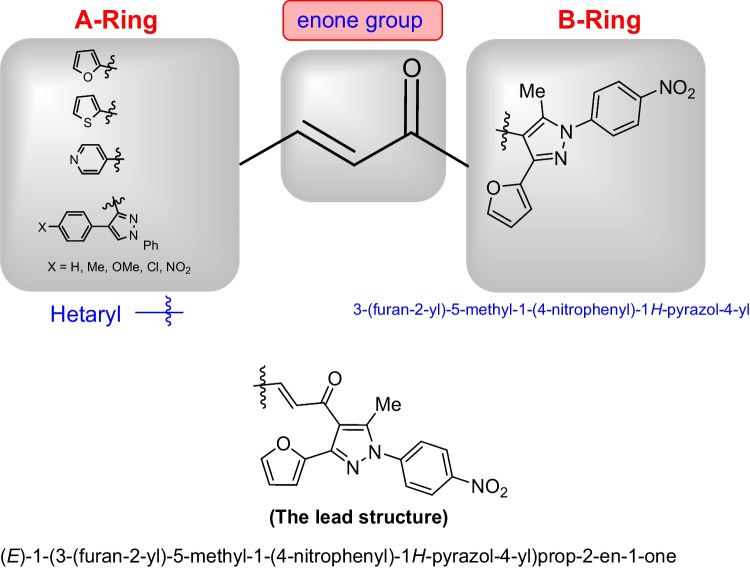
Structural activity relationship (SAR)

The structure can be seen as *α*,*β*-unsaturated enone group linked to two rings (A-ring and B-ring). A-ring represents the heteroaldehyde part and B-ring represents the acetyl part of chalcone, which is 3-(furan-2-yl)-5-methyl-1-(4-nitrophenyl)-1*H*-pyrazole) group. In all compounds, B-ring and the enone group are fixed. The change is only in A-ring (Scheme 3).

### MTT assay of (3-(furan-2-yl)pyrazol-4-yl)chalcones against lung carcinoma (A549) cell line

**Fig. 1:**
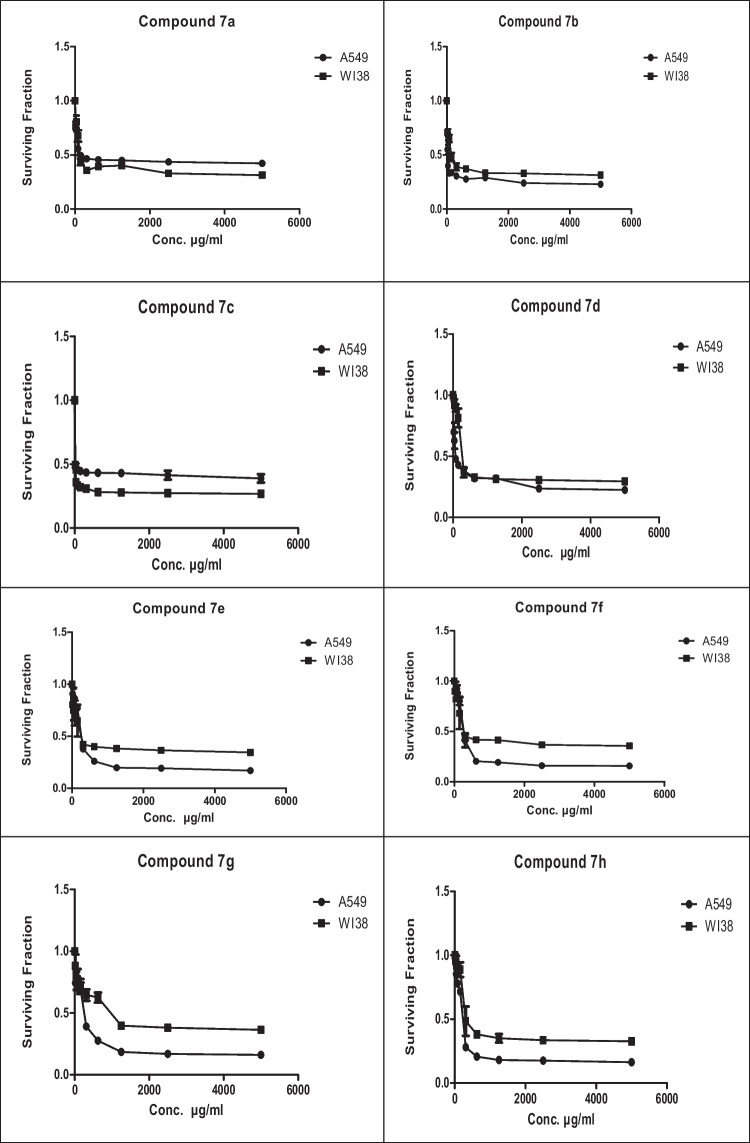
Cytotoxicity of chalcone series **7a-h** against the human A549, and Wi38 cell lines using MTT assay. The cell lines were subjected to different concentrations of novel chalcones **7a-h** for 48 h. All data and standard deviations (SD ± mean) were calculated by the Prism software program (Graph Pad software incorporated, version 3)

Chemotherapeutics are more effective in the case of lung cancer that spread outside the affected area like bones, liver, or adrenal gland. Chemotherapy is not favored in the case of patients with poor health. This experimental part aims to throw light on some of the newly interested groups that were added to the lead structure ((*E*)-1-(3-(furan-2-yl)-5-methyl-1-(4-nitrophenyl)-1*H*-pyrazol-4-yl)prop-2-en-1-one) and their enhanced cytotoxic effect toward lung carcinoma (A549) cell line. Herein, MTT assay has been done to investigate the cytotoxic effect of the target chalcone against lung cancer (A549) and normal lung (Wi38) cell lines. The authors started the work in this paper on the newly prepared compounds by investigating the cytotoxic effect against lung cancer (A549) and normal lung (Wi38) cell lines. The results proved that all tested chalcones give different cytotoxic effects toward lung carcinoma. Activity scales of all tested compounds varied from high to moderate scale, which indicated that our new additions into the lead structure were promoted in this recent study. Generally, as shown in Table 1, it was observed that chalcone **7c** exhibited the best cytotoxic activity among all tested chalcones with IC_50_ value (13.86 µg/ml). This may be due to the additional pyridin-4-yl group in A-ring. Chalcone **7b** also demonstrated high efficiency as cytotoxic effect toward lung carcinoma with IC50 (20 µg/ml). This was due to the presence of thiophen-2-yl group. Next, chalcone **7a** recorded a high cytotoxic effect toward lung cancer with IC50 (42.7 µg/ml) due to the newly added furan-2-yl group. In addition, chalcone **7d** contains the strong cytotoxic 1,4-diphenyl-1*H*-pyrazol-3-yl group which is responsible for the promising effect against the A549 cell line (76.5 µg/ml). Thus, as can be seen in Fig. 1 the most effective and promising cytotoxic activity can be ordered as follows: pyridin-4-yl ˃ thiophen-2-yl ˃ furan-2-yl ˃ 1,4-diphenyl-1*H*-pyrazol-3-yl. On the other hand, it was noted that chalcones containing substituted pyrazole groups (1-phenyl-4-aryl-1*H*-pyrazol-3-yl) as **7 h**, **7f**, **7 g**, and **7e** illustrated moderate cytotoxicity toward lung cancer with IC50 values (211.30, 237.84, 251.49, and 217.01 µg/ml) respectively. Regarding the novel additions within the pyrazole ring itself (in A-ring), it was noted that the pyrazole ring containing either electron-withdrawing or electron-donating groups on the aryl group that exists at position-4 as in compounds **7e**-**7 h** decreased the cytotoxic activity compared to the unsubstituted ph ring as illustrated in chalcone **7d**. In addition, it was demonstrated that chalcone **7 h** containing 1-phenyl-4-(*p*-tolyl)-1*H*-pyrazol-3-yl group enhanced the cytotoxic effect rather than **7f** that had 4-(4-methoxyphenyl)-1-phenyl-1*H*-pyrazol-3-yl. On the other hand, 4-(4-nitrophenyl)-1-phenyl-1*H*-pyrazol-3-yl group in chalcone **7e** enhanced the biological effect more than 4-chlorophenyl)-1-phenyl-1*H*-pyrazol-3-yl in compound **7 g**. At the end of this session, all chalcones derivatives were tested against healthy lung cells (Wi38) and showed a variable toxic effect. Chalcone **7 g** illustrated the least toxic effect toward normal lung cell line with IC50 value (379.22 µg/ml). Chalcones **7f**, **7e**, **7d**, and **7 h** showed less toxic activity with IC50 values (296.24, 295.59, 261.32, and 224.36 µg/ml) respectively, while chalcone **7b** displayed moderate toxic effect with IC50 (108.41 µg/ml). Both chalcones **7a** and **7c** indicated the high toxic effect toward normal lung cells with IC50 values (68 and 18.2 µg/ml) respectively (Fig. 1).

**Table 1:** MTT assay of different (3-(furan-2-yl)pyrazol-4-yl)chalcone compounds against lung carcinoma and normal lung cell lines using 5-FU as a positive control

IC50 values (µg/ml)									
	**7a**	**7b**	**7c**	**7d**	**7e**	**7f**	**7g**	**7h**	5-FU
Lung carcinoma (A549)	42.70	20.05	13.86	76.52	217.01	237.84	251.49	211.30	147.29
Normal lung (Wi38)	68	108.41	18.2	261.32	295.59	296.24	379.22	224.36	__

**Fig. 2:**
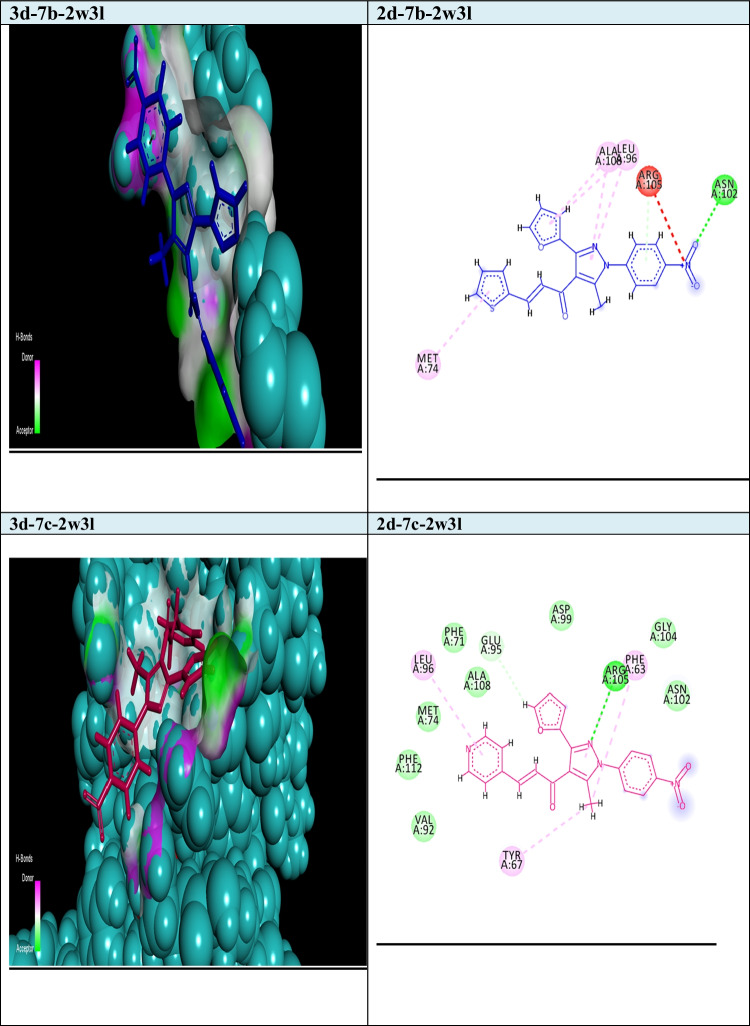
2D and 3D modelling representations for chalcones 7b and 7c against 2w3l active domain

## Molecular docking

**Fig. 3:**
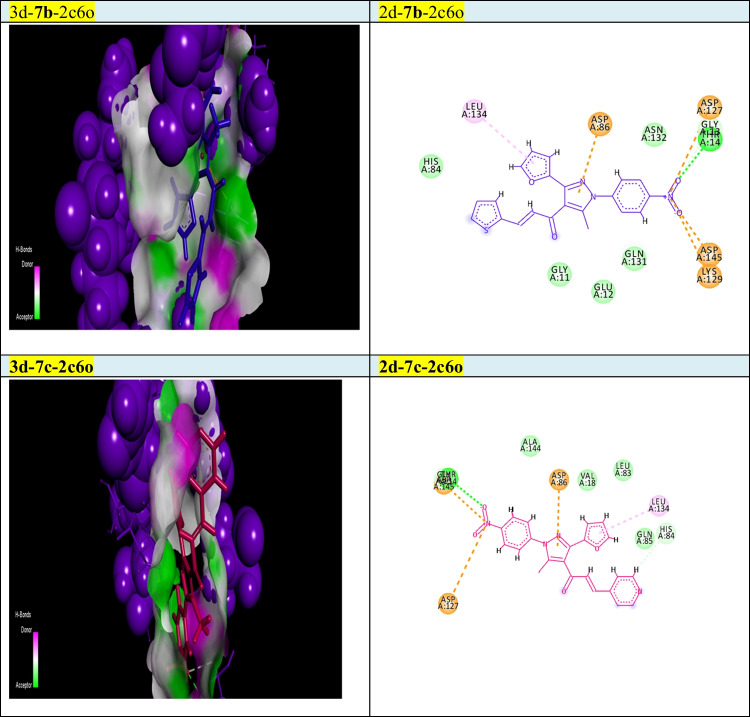
2D and 3d modelling representations for chalcones **7b** and **7c** against 2c6o active domain

**Fig. 4:**
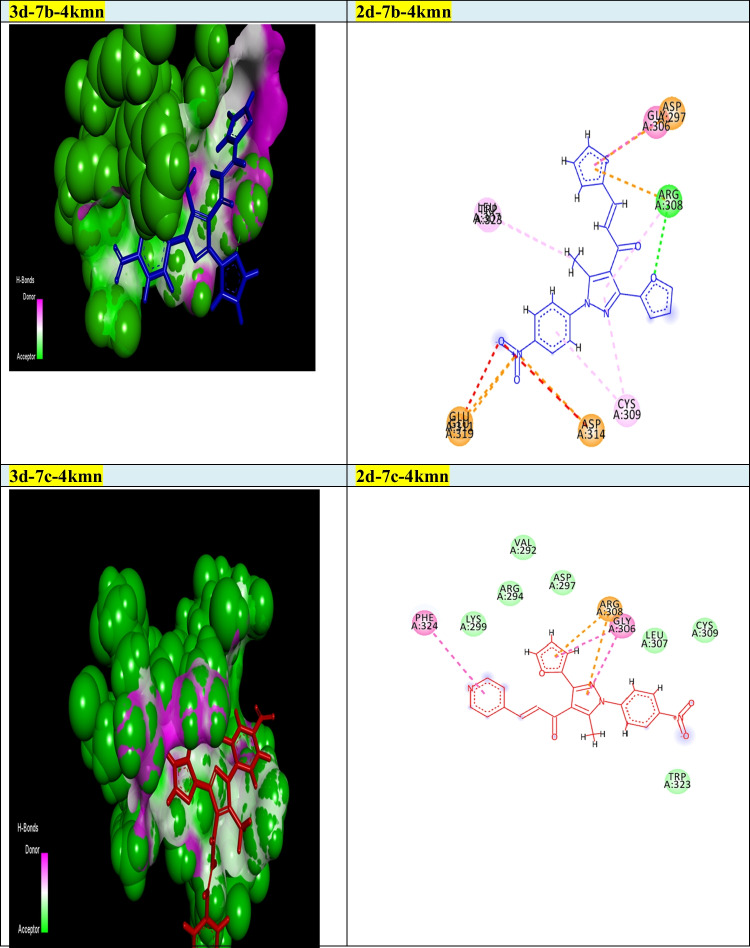
2D and 3D modelling representations for chalcones **7b** and **7c** against 4kmn active domain

**Fig. 5:**
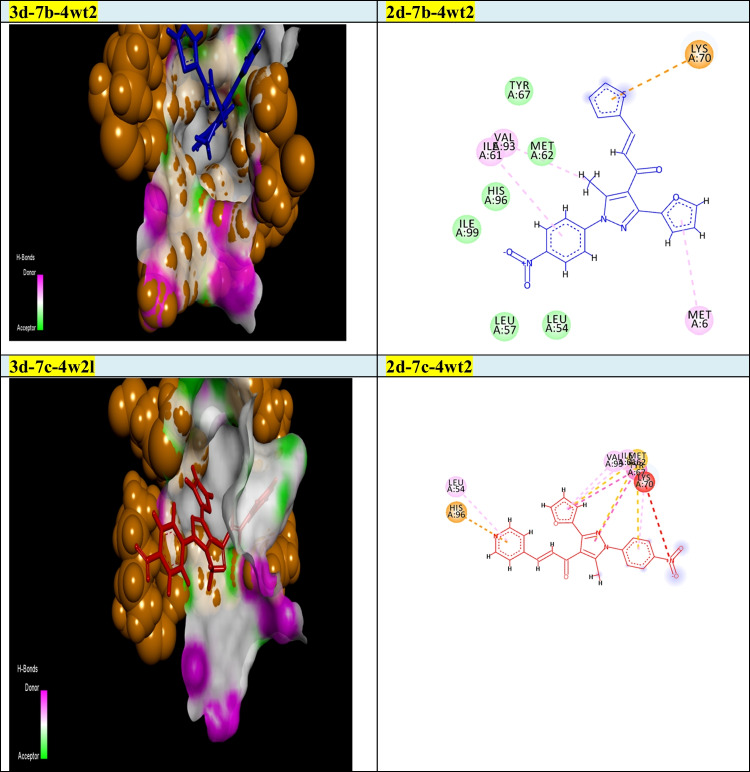
2D and 3D modelling representations for chalcones **7b** and **7c** against 4wt2 active domain

**Fig. 6:**
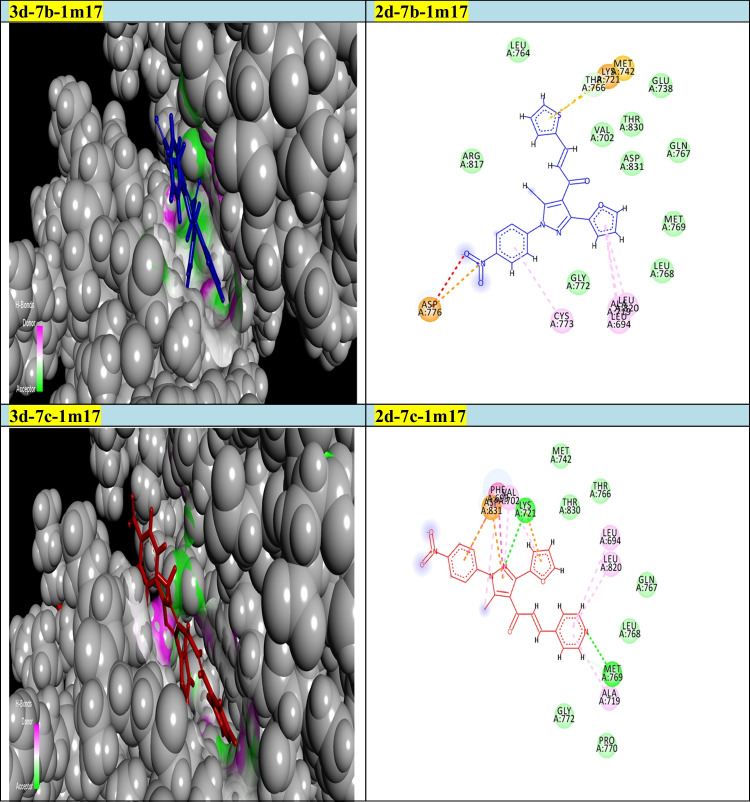
2D and 3D modelling representations for chalcones 7b and 7c against 1m17 active domain

Our goal in this simulation study is to develop a promising irreversible inhibitor with large selectivity and binding affinity toward the active centers of the tested protein markers from the database. The compounds model designed in this study was primarily validated to check for the enhanced and promised model that can identify the active compounds in a virtual screening process against different protein marker sets. Current modelling studies on the most active two chalcones **7b** and **7c** were focused on their inhibition and binding affinities toward the protein domains that help cancer cells to resist and propagate as (2w3l, 2c6o, 4kmn, 1m17, and 4wt2) active domains (Figs. 2-6). In this work and related references (Stamos et al. 2002; Berman et al. 2003; Porter et al. 2009; Tantraworasin et al. 2013), it was observed that the same standard inhibitors co-crystallized with selected domains were utilized as reference ligands for comparison with our new prepared compounds. The tested two chalcones **7b** and **7c** indicated different binding modes into the target domains with variable energies of binding. For a visual representation of the degree of binding affinities of each chalcone to the active sites, authors were referred to energy readings of each compound respectively (Tables 3 and 4). On the other hand, the binding energies of standard inhibitor complexed with each domain were demonstrated in Table 2. In general, the two chalcones showed enhanced binding affinities against all tested protein markers relative to standard inhibitors. In a specific way, chalcone **7c** was the most promising compound and illustrated strong affinities toward 1m17 domain as showed in Fig. 6. The amino acids blocked by our target new chalcone **7c** were MET A:769, LEU A:820, LEU A:694, LYS A:721, VAL A:702, PHE A:698, and ASP A:83. In addition, the chalcone **7c** achieved the best affinity with Gibbs free energy (− 24.5 kcal/mol) that proposed a high binding affinity more than chalcone **7b** (− 23.7 kcal/mol) relative to that of standard ligand (− 23.9 kcal/mol) against 1m17 protein. In addition, chalcone **7c** offered enhanced binding affinities towards the two active domains 2c6o and MDM2 (4wt2)(with specific binding affinities (− 23.33, − 21.86 kcal/mol) respectively (Table 3). Compared with the data shown in Table 2, standard inhibitor energy readings for both domains 2c6o and 4wt2 were − 26.7 and − 14.3 kcal/mol respectively, which indicated strong binding affinity of chalcone **7c** toward 4wt2 domain and moderate effect toward the other domain 2c6o. As illustrated in Fig. 3, the amino acids involved in the active site of 2c6o and blocked by chalcone **7c** were ASP A:127, ASP A:86, LEU A:134, and GLY A:145. On the other hand, Fig. 5 showed the amino acids of the active site of 4wt2 that blocked by chalcone **7c** were LEU A:54, HIS A:96, LYS A:70, MET A:62, VAL A93, and TYR A:67. On the other hand, regarding the two active domains Bcl2-xl (2w3l) and cIAP1-BIR3 (4kmn), we found − 19.27, and − 19.77 kcal/mol. It was noted that, chalcone **7c** still proposed strong and promising effect in comparison with standard ligand values (− 18.3, − 14.4 kcal/mol). As outlined in Figs. 2 and 4, the amino acids blocked by chalcone **7c** with respect to both domains were PHE A:324, ARG A:308, and GLY A:306 for 4kmn and (ARG A:105, PHE A:63, TYR A:67, LEU A:96, and GLU A:95) for 2w3l respectively,

**Table 2:** Energy readings of the standard ligand with different active domains (2c6o, 2wt3, 4kmn, 4wt2, and 1m17). *S* Gibbs free energy, *RMSD* root mean squared deviation, *E* energy

*Kcal/mol*					
	2c6o	2w3l	4kmn	4wt2	1m17
S	− 26.7	− 18.3	− 14.4	− 14.3	− 23.7
rmsd	0.82	4.6	4.9	1.06	1.5
E_place	− 90.3	− 44.3	− 77.5	− 79.7	− 57.2
E_score	− 11.3	− 9.7	− 9.4	− 11.9	− 10.3

**Table 3:** Energy readings of compound **7b** with different active domains (2c6o, 2wt3, 4kmn, 4wt2, and 1m17). *S* Gibbs free energy, *RMSD* root mean squared deviation, *E* energy

*Kcal/mol*					
	2c6o	2w3l	4kmn	4wt2	1m17
S	− 23.87	− 19.43	− 22.12	− 22.42	− 23.99
rmsd	3.56	1.08	2.15	2.76	3.1
E_place	− 72.42	− 58.56	− 40.48	− 61.07	− 56.97
E_score	− 11.46	− 9.43	− 7.67	− 9.05	− 11.01

Regarding chalcone **7b**, it proposed a high binding affinity toward 1m17 domain with binding affinity (− 23.99 kcal/mol) relative to standard inhibitor (− 23.7 kcal/mol). Related to chalcone **7b**, it achieved the best and comparable affinity toward 2c6o with Gibbs free energy (− 23.87 kcal/mol) relative to 1m17 domain. As shown in Fig. 6, the amino acids included in the binding process of chalcone **7b** against 1m17 protein were ASP A:776, CYS A:773, LEU A:694, LEU A:768, ALA A:719, THR A:760, LYS A:721, and MET A742. As exposed in Fig. 3, chalcone **7b** inhibited the active site of 2c6o through the following amino acids (PHE A:14, ASP A:127, ASP A:145, LYS A:129, ASP A:86, and LEU A:134).

In addition, **7b** illustrated a promising and similar affinities toward 4wt2 domain (− 22.42 kcal/mol) and 4kmn (-22.12 kcal/mol) in comparison to positive control inhibitor (− 14.3, and − 14.4 kcal/mol) respectively, As illustrated in Fig. 5, the amino acids of 4wt2 involved in the interactions with chalcone **7b** were LYS A:70, MET A:6, ILE A:61, and TRP A:323, where the amino acid included in the blocking process of chalcone **7b** against 4kmn domain were GLU A:306, ASP A:297, ARG A:308, CYS A:309, ASP A:314, GLU A:319, and TRP A:328. Additionally, chalcone **7b** illustrated good binding affinity toward 2w3l protein with binding energy (− 19.4 kcal/mol) in comparison to co-crystallized standard inhibitor (− 18.3 kcal/mol). As exposed in Fig. 2, the amino acid of 2w3l domain that inhibited by chalcone **7b** were ASN A:102, ARG A:105, LEU A:96, ALA A:108, and MET A:74. Concerning both chalcone **7c** and **7b**, the best binding affinity was achieved toward the 1m17 domain with binding energy (− 24.53, and − 23.99 kcal/mol) respectively, relative to co-crystallized standard inhibitor (− 23.7 kcal/mol). With respect to 4wt2 domain, chalcone **7b** ensured better binding affinity (− 22.42 kcal/mol) than chalcone 4c (− 21.86 kcal/mol). In addition, the two chalcones **7b** and **7c** proved the best binding affinity toward 4wt2 domain in comparison to standard inhibitor ligand (− 14.3 kcal/mol). It was clearly noted that chalcone **7b** still offered the higher binding affinities more than chalcone **7c** regarding the three domains 4kmn, 2w3l, and 2c6o with interaction force (− 22.12, − 19.43, − 23.8 kcal/mol) respectively, for chalcone **7b** and (− 19.77, − 19.27, and − 23.33 kcal/mol) respectively for chalcone **7c** as noted in Tables 3 and 4. Finally, the authors suggested and concluded that theoretical studies proved the new additions and modifications performed in chalcones **7b** and **7c** enhanced their binding affinities toward active sites of protein sets and thus improving the inhibitory effect and death of cancer cells.

**Table 4:** Energy readings of compound **7c** with different active domains (2c6o, 2wt3, 4kmn, 4wt2, and 1m17). *S* Gibbs free energy, *RMSD* root mean squared deviation, *E* energy

*Kcal/mol*					
	2c6o	2w3l	4kmn	4wt2	1m17
S	− 23.33	− 19.27	− 19.77	− 21.86	− 24.53
rmsd	4.325	1.76	3.48	3.34	1.96
E_place	− 70.46	− 74.25	− 74.74	− 30.97	− 61.36
E_score	− 11.653	− 9.11	− 8.36	− 9.39	− 11.03

## The effect of chalcones 7b and 7c on genes expression

**Fig. 7:**
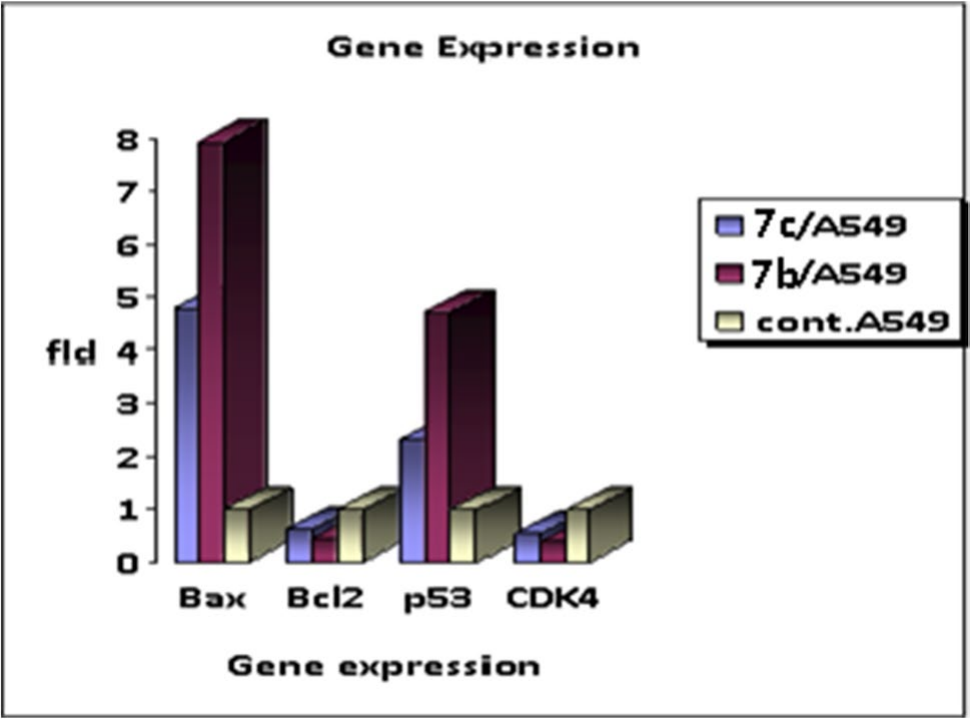
Bar graph represents the relative expression levels of apoptotic genes (BAX and P53) and anti-apoptotic genes (Bcl2 and CDK4) as measured by qPCR. A549 cells treated with compound **7b** or compound **7c** or left untreated were lysed and the extracted total RNA was reverse transcribed into cDNA. The fold expression change was determined by the 2^−ΔΔCt^ method after normalization to the expression level of β-actin as a reference gene

The expression level of the apoptotic genes (Bax and P53) and the anti-apoptotic genes (Bcl2 and CDK4) was studied in A549-treated cells. A549 cells were treated with the IC_50_concentration of compounds **7b** and **7c** (20.05 and 13.86 µg/mL respectively) for 48 h and then subjected to real-time PCR. The untreated A549 cells were used as a negative control. As seen from Table 5 and Fig. 7, for compound **7c**-treated cells, the apoptotic genes P53 and Bax were upregulated with fold change equalled 2.32 and 4.78 respectively relative to the negative control. Regarding the anti-apoptotic genes Bcl2 and CDK4, the expression level was downregulated by about a half as compared to the negative control (fold change = 0.61 and 0.54 respectively). A comparable result was seen for compound **7b**-treated A549 cells. It was found that the expression level of P53 and Bax was increased almost twofold to that of compound **7c** treated cells (fold change = 4.69 and 7.93 respectively). The expression level of Bcl2 and CDK4 was significantly decreased with fold change equalled 0.44 and 0.39 respectively. It was notable that compound **7b** enhanced the expression of the apoptotic genes and lowered the expression of the anti-apoptotic genes more than compound **7c**. It was reported that 3,4-disubstituted pyrazole analogues, and 3-(imidazol-2-yl)-4-[2(pyridin-3-yl)-vinyl]-pyrazoles have been acted as cyclin-dependent kinase (CDK) inhibitors and showed anticancer activity against various cancer cell lines (Hawash et al. 2017). So that the downregulation of CDK4 may be due to the effect of the pyrazole moiety of our compounds. It was found that the substituted chalcone- pyrazole hybrid (2-((3-(4-Methoxyphenyl)-1-phenyl-1*H*-pyrazol-4-yl)methylene)-2H-indene-1,3-dione) showed promising activity against MCF‐7, HepG2, and HCT116 cancer cell lines, and induced apoptosis in breast cancer cells through the upregulation of tumor suppressor genep53 (Gao et al. 2020). So this hybridization showed enhanced activity, not a deleterious one.

**Table 5:** The relative expression levels of BAX,P53, Bcl2, and CDK4 genes in A549 cells after the treatment with IC_50_ conc. of compound **7b** and compound **7c**, untreated A549 cells were used as a negative control

Gene expression (fold change/ β-actin)				
Sample	BAX	Bcl2	P53	CDK4
Compound **7b**/ A549	7.93	0.44	4.69	0.39
Compound **7c**/A549	4.78	0.61	2.32	0.54
Control (A549)	1	1	1	1

## ELISA assay for chalcones 7b and 7c

Apoptosis is a programmed cell death that occurred inside the cells under physiological and pathological conditions. There are two pathways of the apoptosis process, mitochondrial (intrinsic) pathway and death receptor (extrinsic) pathway (Fulda and Debatin 2006).Caspase-8 is activated in the extrinsic pathway upon stimulation of death receptor which results in the activation of effector caspase-3 (Fulda and Debatin 2006). While, in the intrinsic pathway, caspase-9 participates in the formation of the cytochrome c/Apaf-1/caspase-9 apoptosome complex results in the activation of caspase-3 (Saelens et al. 2004). Caspase-3 is responsible for the breakdown of proteins and DNA resulting in cell death (Degterev et al. 2003). The activity of caspase-3, caspase-8, and caspase-9 in A549 cells after 48 h of treatment with the IC_50_ concentration of compounds **7b** and **7c** was determined using the sandwiched ELISA assay (Table 6). It was found that compounds **7b** and **7c** increased the expression level of the studied caspase-3, caspase-8, and caspase-9. For caspase-3, both compounds enhanced greatly the activity (495.4 and 377.2 pg/mL for compounds **7b** and **7c** respectively) relative to the control (53.69 pg/mL), while the concentration of caspase-8 was slightly increased in response to compounds **7b** and **7c** (1.575and 1.042 pg/mL respectively) relative to the negative control (0.375 pg/mL). Compounds **7b** and **7c** upregulated the expression level of caspase-9 moderately (15.76and 11.2 pg/mL respectively) as compared to the negative control (1.49 pg/mL). It was noticed that compound **7b** had a higher effect on the activity of the studied caspases than compound **7c**. So from these results, compounds **7b** and **7c** induced both extrinsic and intrinsic pathways of apoptosis.

**Table 6:** The determination of the expression level of caspase-3, caspase-8, and caspase-9 in A549 cells using the Enzyme-linked immune sorbent assay (ELISA) technique. The untreated cells were used as a negative control. Data represented the mean ± SD

Enzyme activity (pg/mL) ± SD			
Sample	Caspase 3	Caspase 8	Caspase 9
Compound 7b/A549	495.4 ± 6.52	1.575 ± 0.05	15.76 ± 0.06
Compound 7c/A549	377.2 ± 6.04	1.042 ± 0.02	11.2 ± 0.52
Control (A549)	53.69 ± 2.91	0.375 ± 0.034	1.49 ± 0.04

## Flow-cytometric analysis of cell cycle and apoptosis

**Fig. 8:**
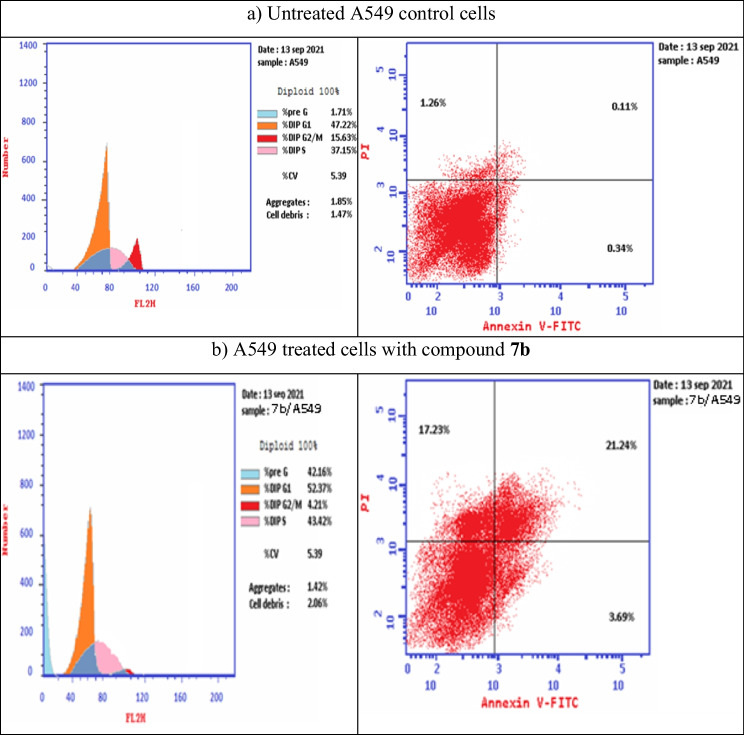
Flow cytometric analysis of cell cycle (left-handed side) and apoptosis (right-handed side) of untreated and treated lung cancer cells. **A** Control sample of untreated A549 cells. **B** Treated A549 cells with compound **7b** after 48 h of treatment. The percentage and distribution of cells in the different phases of the cell cycle are indicated. The untreated cells were used as a negative control

As shown from the above molecular studies, compound **7b** demonstrated higher activity than compound **7c**, suggesting that the thiophene was better than the pyridine ring. So, the flow cytometric analysis was done on the most active compound **7b**. The cell cycle is regulated by several proteins called cyclins and their associated serine/threonine cyclin-dependent kinases (CDKs) (Baker and Reddy 2012). Among these CDKs, CDK4 mediates the transition of cells from the G1 to S phase. As mentioned in the RT-PCR section, that compound **7b** inhibited the expression of CDK4. This result suggested the accumulation of cells at the G1 phase which was already happened in our flow cytometric analysis of the cell cycle. As shown in Fig. 8 and Table 7, compound **7b** caused cell cycle arrest at G0/G1 and S phases. Regarding the flowcytometric analysis of apoptosis, it was noticed that compound **7b** induced apoptosis in A549-treated cells. Figure 8 and Table 8 indicated that the percentage of early apoptotic cells was 3.69% relative to the untreated control cells (0.34%). The percentage of late apoptotic cells was significantly increased (21.24%) as compared to the control cells (0.11%). Also, it was found that compound **7b** induced necrosis process by a percentage of 17.23% relative to the control cells (1.26%). By comparing our results with the results of other different chalcone hybrids from literature, it was found that the chalcone-pyridine hybrid *(E)-1-(2,6-dimethoxypyridin-4-yl)-3-(3-hydroxy-4 methoxyphenyl)-2-methylprop-2-en-1-one* caused cell cycle arrest at G2/M phase and induced cell apoptosis (Xu et al. 2019). Chalcone-furan hybrid *3-[4-(dimethylamino)phenyl]-1-(7-ethoxy-1-benzofuran-2-yl)prop-2-en-1-one* could induce apoptosis through caspase-dependent pathways in prostate, lung, and breast cancer cells (Coskun et al. 2017). It was reported that the structural activity relationship (SAR) indicated that hybrids with thiophene moiety were more active than the corresponding furan analogs (Gao et al. 2020). The anticancer SAR of chalcone‐1,4‐dihydroindeno[1,2‐c]pyrazole hybrids could arrest the cell cycle of the A549 cell line in the G2/M phase (Khan et al. 2019).

**Table 7:** The percentage of DNA content in the different phases of the cell cycle of treated A549 cells with compound **7b** relative to the untreated control A549 cells

Sample	G0/G1%	S %	G2/M %
Control (A549)	47.22	37.15	15.63
Compound **7b/**A549	52.37	43.42	4.21

**Table 8:** The flow cytometric analysis of the percentage of apoptosis and necrosis in A549 cells treated with compound **7b**following 48 h of treatment relative to the untreated lung cancer cells

Samples	Apoptosis %			Necrosis%
	%Total	% Early	% Late	
Control (A549)	1.71	0.34	0.11	1.26
Compound **7b**/A549	42.16	3.69	21.24	17.23

## Experimental part

### Chemistry

Melting points were measured with a Stuart melting point apparatus and are uncorrected. The IR spectra were recorded using a FTIR Bruker–vector 22 spectrophotometer as KBr pellets. The ^1^H and ^13^C NMR spectra were recorded in CDCl_3_ and DMSO as solvents on Varian Gemini NMR spectrometer at 300 MHz using TMS as internal standard. Chemical shifts are reported as *δ* values in ppm. Mass spectra were recorded with a Shimadzu GCMS–QP–1000 EX mass spectrometer in EI (70 eV) model. The elemental analyses were performed at the Microanalytical Center, Cairo University.

### Synthesis of (E)-3-heteroaryl-1-(3-(furan-2-yl)-5-methyl-1-(4-nitrophenyl)-1H-pyrazol-4-yl)prop-2-en-1-one derivatives (7a-h)

To a stirred mixture of 4-acetyl-1-(4-nitrophenyl)-1*H*-pyrazole **5** (0.001 mol) and the appropriate heteroaldehydes **6** (0.001 mol) in ethanol (30 ml), sodium hydroxide solution 20% was added, and the reaction mixture was stirred for 6 h at room temperature and left overnight. The resulting solid product that precipitated was filtered, washed with water, and crystallized from a suitable solvent to give the corresponding chalcones **7a-h**.

### (E)-3-(Furan-2-yl)-1-(3-(furan-2-yl)-5-methyl-1-(4-nitrophenyl)-1H-pyrazol-4-yl)prop-2-en-1-one (7a)


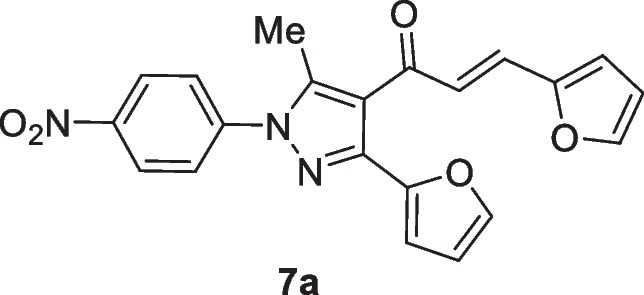


Yellow crystals, mp 138–140 °C (EtOH-Dioxane), Yield (80%); IR (*ν*_max_, cm^−1^) *ν* 1659 (CO). ^1^H NMR (300 MHz, CDCl_3_) *δ* 2.60 (s, 3H, CH_3_), 6.47–6.49 (m, 2H, furan-H), 6.65 (d, 1H, furan-H, *J* = 3.3 Hz), 6.84 (d, 2H, furan-H, *J* = 3.6 Hz), 6.86 (d, 1H, vinyl-H, *J* = 16.2 Hz), 7.42 (m, 1H, furan-H, *J* = 3.6 Hz), 7.47 (d, 1H, vinyl-H, *J* = 16.2 Hz), 7.74 (d, 2H, Ar–H, *J* = 9 Hz), 8.37 (d, 2H, Ar–H, *J* = 9 Hz); ^13^C NMR (75 MHz, CDCl_3_) *δ* 12.6, 111.0, 111.3, 112.5, 115.9, 121.0, 123.5, 124.6, 125.4, 129.5, 142.8, 143.1, 143.4, 143.6, 144.9, 145.9, 146.7, 151.1, 186.9. MS (EI, 70 eV) m/z (%): 389 (M^+^). Anal. Calcd. for C_21_H_15_N_3_O_5_ (389.37): C, 64.78; H, 3.88; N, 10.79. Found: C, 64.91; H, 3.97; N, 10.93.

### (E)-1-(3-(Furan-2-yl)-5-methyl-1-(4-nitrophenyl)-1H-pyrazol-4-yl)-3-(thiophen-2-yl)prop-2-en-1-one (7b)


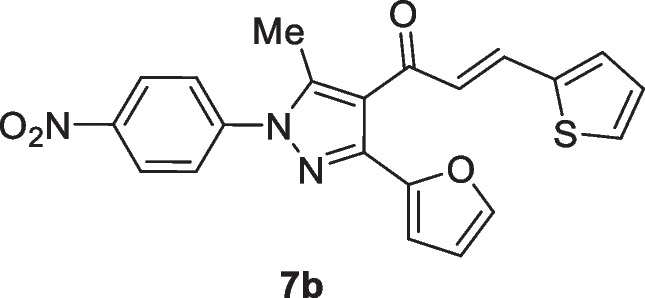


Yellow crystals, mp 142–144 °C (EtOH-Dioxane), Yield (85%); IR (*ν*_max_, cm^−1^) *ν* 1658 (CO). ^1^H NMR (300 MHz, CDCl_3_) *δ* 2.61 (s, 3H, CH_3_), 6.51–6.59 (m, 1H, furan-H), 6.74 (d, 1H, vinyl-H, *J* = 15.3 Hz), 6.83 (d, 1H, thiophene-H, *J* = 3.3 Hz), 7.04–7.07 (m, 1H, thiophene-H), 7.26 (d, 1H, thiophene-H, *J* = 2.7 Hz), 7.38 (d, 1H, furan-H, *J* = 4.8 Hz), 7.55 (m, 1H, furan-H), 7.74 (d, 2H, Ar–H, *J* = 9 Hz), 7.81 (d, 1H, vinyl-H, *J* = 15.3 Hz), 8.40 (d, 2H, Ar–H, *J* = 9 Hz); ^13^C NMR (75 MHz, CDCl_3_) *δ* 12.6, 111.1, 111.3, 120.9, 124.5, 124.7, 125.3, 128.2, 128.8, 131.6, 135.4, 139.9, 143.20, 143.27 143.31, 143.6, 145.8, 146.7, 186.4. MS (EI, 70 eV) m/z (%): 405 (M^+^). Anal. Calcd. for C_21_H_15_N_3_O_4_S (405.43): C, 62.21; H, 3.73; N, 10.36;. Found: C, 62.32; H, 3.94; N, 10.43.

### (E)-1-(3-(Furan-2-yl)-5-methyl-1-(4-nitrophenyl)-1H-pyrazol-4-yl)-3-(pyridin-4-yl)prop-2-en-1-one (7c)


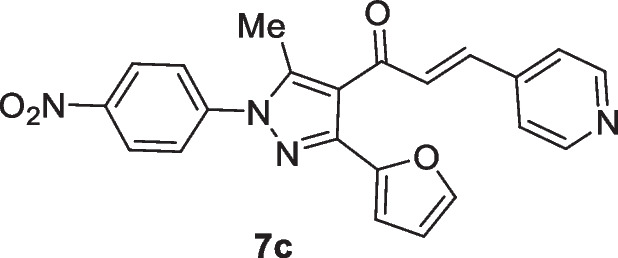


Yellow crystals, mp 198–200 °C (EtOH-Dioxane), Yield (82%); IR (*ν*_max_, cm^−1^) *ν* 1653 (CO). ^1^H NMR (300 MHz, CDCl_3_) *δ* 2.64 (s, 3H, CH_3_), 6.56–6.57 (m, 1H, furan-H), 6.85 (d, 1H, furan-H, *J* = 3.5 Hz), 7.07 (d, 1H, vinyl-H, *J* = 15.9 Hz), 7.25 (d, 2H, pyridine-H), 7.53 (d, 1H, vinyl-H, *J* = 15.9 Hz), 7.58 (m, 1H, furan-H), 7.74 (d, 2H, Ar–H, *J* = 9 Hz), 8.43 (d, 2H, Ar–H, *J* = 9 Hz), 7.38 (d, 2H, pyridine-H, *J* = 4.5 Hz); ^13^C NMR (75 MHz, CDCl_3_) *δ* 12.8, 111.1, 111.6, 121.7, 124.6, 125.4, 125.5, 129.5, 139.4, 141.8, 143.1, 143.8, 144.1, 145.7, 146.9, 149.3, 150.3, 186.2. MS (EI, 70 eV) m/z (%): 400 (M^+^). Anal. Calcd. for C_22_H_16_N_4_O_4_ (400.39): C, 66.00; H, 4.03; N, 13.99;. Found: C, 66.14; H, 4.11; N, 14.12.

### (E)-3-(1,4-Diphenyl-1H-pyrazol-3-yl)-1-(3-(furan-2-yl)-5-methyl-1-(4-nitrophenyl)-1H-pyrazol-4-yl)prop-2-en-1-one (7d)


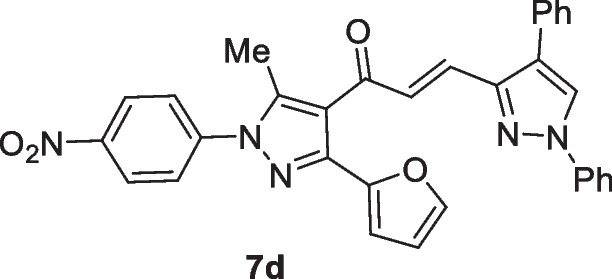


Yellow crystals, mp 168–170 °C (Dioxane-EtOH), Yield (92%); IR (*ν*_max_, cm^−1^) *ν* 1652 (CO). ^1^H NMR (300 MHz, CDCl_3_) *δ* 2.57 (s, 3H, CH_3_), 6.54–6.56 (m, 1H, furan-H), 6.79 (d, 1H, vinyl-H, *J* = 15.3 Hz), 6.85 (d, 1H, furan-H, *J* = 2.7 Hz), 7.35–7.78 (m, 14H, Ar–H + furan-H + vinyl-H), 8.15 (s, 1H, pyrazole-H5), 8.40 (d, 2H, Ar–H, *J* = 9 Hz); ^13^C NMR (75 MHz, CDCl_3_) *δ* 12.5, 110.9, 111.5, 117.8, 119.1, 120.9, 124.6, 125.4, 125.7, 126.4, 127.2, 128.51, 128.57, 128.61, 129.4, 131.9, 134.5, 139.2, 142.9, 143.2, 143.40, 143.47, 146.2, 146.8, 153.6, 187.6. MS (EI, 70 eV) m/z (%): 541 (M^+^). Anal. Calcd. for C_32_H_23_N_5_O_4_ (541.57): C, 70.97; H, 4.28; N, 12.93; Found: C, 71.12; H, 4.34; N, 12.78.

### (E)-1-(3-(Furan-2-yl)-5-methyl-1-(4-nitrophenyl)-1H-pyrazol-4-yl)-3-(1-phenyl-4-(p-tolyl)-1H-pyrazol-3-yl)prop-2-en-1-one (7e)


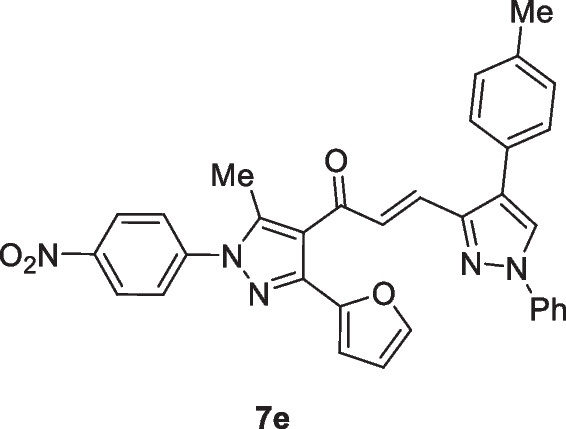


Yellow crystals, mp 248–250 ^ο^C (Dioxane-EtOH), Yield (87%); IR (*ν*_max_, cm^−1^) *ν* 1652 (CO). ^1^H NMR (300 MHz, CDCl_3_) *δ* 2.61 (s, 3H, CH_3_), 2.9 (s, 3H, CH_3_), 6.57–6.58 (m, 1H, furan-H), 6.79 (d, 1H, vinyl-H, *J* = 16.2 Hz), 6.84 (d, 1H, furan-H, *J* = 3 Hz), 7.40–7.81 (m, 11H, Ar–H + furan-H + vinyl-H), 8.14 (s, 1H, pyrazole-H5), 8.29 (d, 2H, Ar–H, *J* = 8.7 Hz), 8.42 (d, 2H, Ar–H, *J* = 9 Hz). MS (EI, 70 eV) m/z (%): 555 (M^+^). Anal. Calcd. for C_33_H_25_N_5_O_4_ (555.59): C, 71.34; H, 4.54; N, 12.61; Found: C, 71.50; H, 4.63; N, 12.77.

### (E)-1-(3-(Furan-2-yl)-5-methyl-1-(4-nitrophenyl)-1H-pyrazol-4-yl)-3-(4-(4-methoxyphenyl)-1-phenyl-1H-pyrazol-3-yl)prop-2-en-1-one (7f)


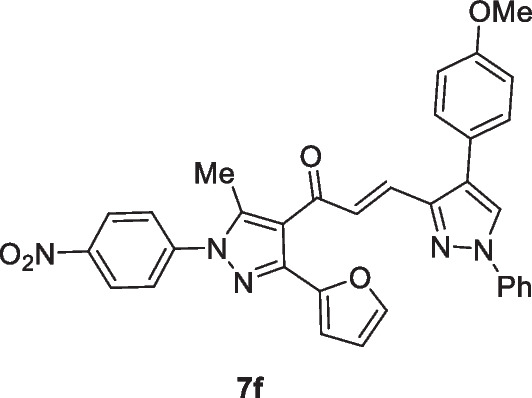


Yellow crystals, mp 232–234 °C (Dioxane-EtOH), Yield (84%); IR (*ν*_max_, cm^−1^) *ν* 1653 (CO). ^1^H NMR (300 MHz, CDCl_3_) *δ* 2.57 (s, 3H, CH_3_), 3.87 (s, 3H, OCH_3_), 6.55–6.56 (m, 1H, furan-H), 6.78 (d, 1H, vinyl-H, *J* = 16.2 Hz), 6.85 (d, 1H, furan-H, *J* = 3.6 Hz), 6.97 (d, 2H, Ar–H, *J* = 8.7 Hz), 7.34–7.72 (m, 9H, Ar–H + furan-H + vinyl-H), 7.75 (d, 2H, Ar–H, *J* = 6.9 Hz), 8.13 (s, 1H, pyrazole-H5), 8.40 (d, 2H, Ar–H, *J* = 8.7 Hz); ^13^C NMR (75 MHz, CDCl_3_) *δ* 12.5, 55.3, 111.1, 111.5, 114.1, 117.7, 119.2, 120.9, 124.5, 124.7, 125.5, 126.3, 127.2, 129.5, 129.8, 134.9, 135.8, 135.9, 139.3, 142.9, 143.3, 143.5, 146.3, 146.9, 153.6, 159.9, 187.8. MS (EI, 70 eV) m/z (%): 571 (M^+^). Anal. Calcd. for C_33_H_25_N_5_O_5_ (571.59): C, 69.34; H, 4.41; N, 12.25; Found: C, 69.49; H, 4.58; N, 12.34.

### (E)-3-(4-(4-Chlorophenyl)-1-phenyl-1H-pyrazol-3-yl)-1-(3-(furan-2-yl)-5-methyl-1-(4-nitrophenyl)-1H-pyrazol-4-yl)prop-2-en-1-one (7g)


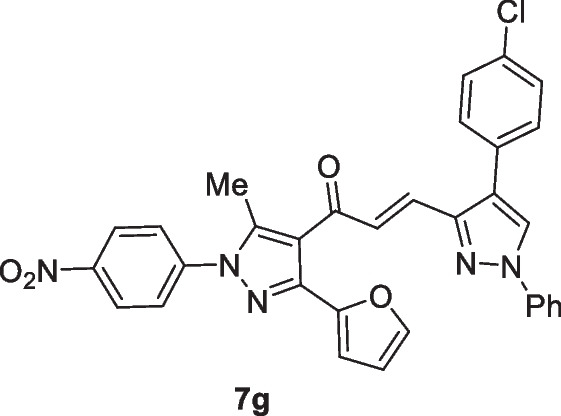


Yellow crystals, mp 242–244 °C (Dioxane-EtOH), Yield (85%); IR (*ν*_max_, cm^−1^) *ν* 1655 (CO). ^1^H NMR (300 MHz, DMSO-d_6_) *δ* 2.50 (s, 3H, CH_3_), 6.61–6.62 (m, 1H, furan-H), 6.88 (d, 1H, furan-H, *J* = 3.6 Hz), 6.95 (d, 1H, vinyl-H, *J* = 15.9 Hz), 7.38–7.90 (m, 11H, Ar–H + furan-H + vinyl-H), 7.94 (d, 2H, Ar–H, *J* = 8.7 Hz), 8.42 (d, 2H, Ar–H, *J* = 8.7 Hz), 9.09 (s, 1H, pyrazole-H5). MS (EI, 70 eV) m/z (%): 576 (M^+^). Anal. Calcd. for C_32_H_22_ClN_5_O_4_ (576.01): C, 66.73; H, 3.85; N, 12.16; Found: C, 66.81; H, 3.98; N, 12.29.

### (E)-1-(3-(Furan-2-yl)-5-methyl-1-(4-nitrophenyl)-1H-pyrazol-4-yl)-3-(4-(4-nitrophenyl)-1-phenyl-1H-pyrazol-3-yl)prop-2-en-1-one (7h)


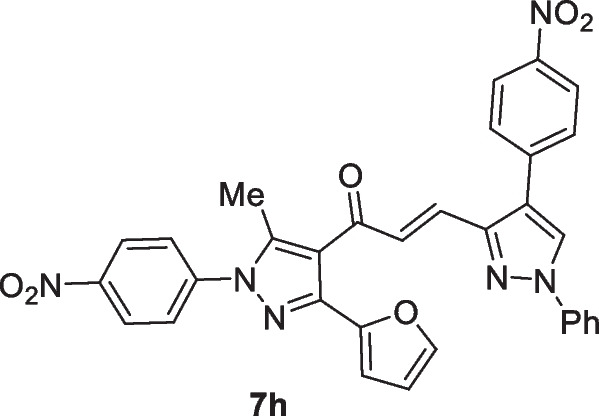


Yellow crystals, mp 242–242 ^ο^C (Dioxane-EtOH), Yield (83%); IR (*ν*_max_, cm^−1^) *ν* 1651 (CO). ^1^H NMR (300 MHz, CDCl_3_) *δ* 2.61 (s, 3H, CH_3_), 6.55–6.56 (m, 1H, furan-H), 6.84 (d, 1H, vinyl-H, *J* = 15.6 Hz), 6.86 (d, 1H, furan-H, *J* = 2.1 Hz), 7.11–7.77 (m, 12H, Ar–H + furan-H + vinyl-H), 7.88 (d, 1H, vinyl-H, *J* = 15.9 Hz), 8.1 (s, 1H, pyrazole-H5), 8.41 (d, 2H, Ar–H, *J* = 9 Hz); ^13^C NMR (75 MHz, CDCl_3_) *δ* 12.7, 111.2, 111.6, 117.8, 119.3, 121.0, 124.8, 125.6, 126.0, 126.3, 126.6, 127.0, 127.4, 127.8, 129.6, 133.7, 133.8, 139.1, 143.2, 143.4, 143.5, 146.3, 147.0, 147.9, 187.5. MS (EI, 70 eV) m/z (%): 586 (M^+^). Anal. Calcd. for C_32_H_22_N_6_O_6_ (586.56): C, 65.53; H, 3.78; N, 14.33; Found: C, 65.67; H, 3.89; N, 14.49.

## Anticancer test

In vitro inhibition activity of the newly synthesized series was investigated on cell viability according to the literature review described by Mohamed et al. (Mohamed et al. 2021b). In a brief explanation of MTT assay employed herein. In vitro, toxicological MTT Kit has been used to perform an (3-[4,5-dimethylthiazole-2-yl]-2,5-diphenyltetrazolium bromide) to measure the cytotoxicity effect of a novel (3-(Furan-2-yl)pyrazol-4-yl) chalcones against A549 cell line. Lung carcinoma cell line A549 was seeded in microplate where the prepared chalcones solution were added to triplicates well. Incubation of plates was done at 37 °C for 48 h. The fresh medium was replaced by the old one. Lung cells treated with different range concentration of our series [100, 50, 25, 12.5, 6.25, 3.125, and 1.56 mg/mL] were incubated, also untreated cells were used as a reference negative control. One percent antibiotic mixture was used, and after incubation. The positive control utilized here was 5-FU (100 mg/mL).

## Molecular docking

A trial was performed to establish 3D model structures of chalcones **7b** and **7c** with different anti-apoptotic protein markers by the same proposal as illustrated by Mohamed et al. (Mohamed et al. 2021b). Based on the crystal structure of selected protein sets under the code access (2w3l, 2c6o, 4kmn, 1m17, and 4wt2), the amino acid sequences were collected from the PDB protein data bank (http://www.RCSB.org/). The 3D structure was established using homology steps implemented by MOE 2010 program. Stable binding energies were achieved by constructing the models and subjecting them to energy minimization 0.05 rms gradient. The final chalcones structures were docked with the active sites of the above-mentioned protein sets. The two synthesized chalcones **7b** and **7c** were sketched using ChemDraw builder and were converted to their perspective 3D form. Subsequently, the energy of compounds was minimized up to 0.05 using the MMFF94x force field. All compounds were docked into the active pocket of all protein groups and conformations of each compound were generated with a docking score. Each compound was analyzed and visualized as a 3D representation according to the BIOVIA Discovery Studio program.

## Real-time PCR

Total RNAfrom the compound **7b-**, compound **7c**-treated A549 cells, and untreated cells were extracted using the Qiagen RNAextraction Kit, Catalog # (74,104). 1 µg of RNAwas reverse transcribed into complementary DNA(cDNA) using High-Capacity cDNA Reverse Transcription Kit (Thermo Fisher scientific, ON, Canada). qPCR was performed using Bio RadSYBR Green qPCR master mix (Bio Rad, California, USA) in a Step One Plus Real-Time PCRSystem (Bio Rad, California, USA). The total reaction volume was 25 µL, 12.5 µL of SYBRgreen master mix, 1 µL of each primer (Bax, Bcl2, P53, CDK4) with 10 pmol/µL (Qiagen, CA, Germany), 2.5 µL of cDNA, and 9 µL of RNAse free water. The thermal cycle of PCR started with an initial denaturation at 95 °C for 10 min, followed by 45 cycles at 94 °C for15 s, 65 °C for 30 s, and 56 °C for 30 s. The relative gene expression was determined using the method of 2^−ΔΔCt^after normalization to the expression of *β*-actin (Qiagen, CA, Germany) (10 pmol/µL). Primer sequences used according to the published literature as follows: Bax5′-ATG GAC GGGTCC GGG GAG-3′ (forward) and 5′-ATCCCC AACAGC CGC-3′ (reverse); for Bcl2 5′ -AAG CCG GCG ACGACT TCT-3′ (forward) and 5′- GGT GCC GGT TCA GGTACT CA-3′ (reverse); for p53 5′-AGAGTCTATAGGCCCACCCC-3′ (forward) and 5′-GCTCGACGCTAGGATCTGAC-3′ (reverse); for CDK45′-CATCGTTCACCGAGATCTGA-3′ (forward) and 5′-CCAACACTCCACAGATCCAC-3′ (reverse); for β-actin 5′-ATC GTG GGG CGC CCC AGGCAC-3′ (forward) and5′-CTC CTTAATGTCACGCACGAT TTC-3′ (reverse).

## ELISA assay

Elisa assay was performed to detect the concentration of human caspase-3, caspase-8, and caspase-9 in A549 cell culture lysates. The instructions were followed up according to the manuscript instructions described in the following kits: DRG® human Caspase-8 ELISA Kit, Catalogue # **(**EIA-4863**)**, DRG® human Caspase-9 ELISA Kit, Catalogue # (EIA-4860), and Invitrogen human Caspase-3 Elisa Kit, Catalogue # KHO1091, respectively. All reagents, samples, and standards were prepared as described in the above ELISA kits. Briefly, the procedure was described as followed; the microwell strips were washed twice with wash buffer. A total of 100 μl of standard or samples were added to each well and incubated for 2 h at room temperature (18 to 25 °C). The microwell strips were emptied and washed three times with wash buffer. A total of 100 μl of prepared antibodyanti-rabbit-IgG-HRP) were added to each well for 1 h at room temperature. The step of washing was repeated three times and then, 100 μl of prepared TMB (tetramethyl-benzidine) substrate solution was pipetted to all wells and incubated for 10 min at room temperature. For complete inactivation of the enzyme, 100 μl of stop solution was added to each well. The absorbance of each microwell was read immediately on a spectrophotometer at a wavelength of 450 nm. The concentrations of the unknown samples and controls were determined from the standard curve which was plotted using curve fitting software.

## Flow-cytometric analysis of cell cycle and apoptosis

10^6^ of A549 cells were cultured in 60-mm Petri dishes for 24 h and then treated with compound **7b**atIC_50_concentrationfor 48 h. The untreated A549 cells were used as a negative control. After 48 h of incubation, A549 cells were centrifuged at1000 rpm for 5 min at 4 °C. The cells pellet were washed in phosphate-buffered saline (PBS) and then centrifuged at 1000 rpm for another 5 min. After discarding the supeRNAtant, the cells were collected in a single cell suspension and fixed in 70% ethanol on ice overnight. After the fixation step, cells were washed with 1 ml 1 × PBS. Finally, the cells pellet was incubated with a 200 µl 1 × propidium iodide (PI) mixture at room temperature in the dark for 30 min. Then, cells were subjected to an Epics XL-MCL flow cytometer (Beckman Coulter, Miami, FL) for DNA content analysis. Cells distribution at different phases was analysed by Multicycle software (Phoenix Flow Systems, San Diego, CA). Annexin V-FITC kit catalogue number (#K101-25) was used to detect the percentage of the apoptotic cells. About 10^6^ of A549 cells were collected by centrifugation. Then, cells were washed in 500 µl of 1 × PBS. The cells were collected by centrifugation and then resuspended in the annexin V incubation reagent. The annexin V incubation reagent comprising of 10 μL binding buffer (10X) + 10 μL propidium iodide + 1 μL annexin V-FITC + 79 μL deionized water. The cells in 100 μL annexin V incubation reagent was incubated in dark at room temperature for 15 min (Ali et al. 2017). Finally, the percentage of apoptotic cells was analyzed by flow cytometry using FITC signal detector (usually FL-1) and PI staining by the phycoerythrin emission signal detector (usually FL-2).

## Conclusion

From the outcome of our investigations, it was possible to conclude that both chalcones **7b** and **7c** offered better and enhanced cytotoxic effects toward the lung cancer cell line (A549). Molecular modelling data revealed promising binding affinities of our new chalcones **7b** and **7c** toward anti-apoptotic protein markers of the following domains (2w3l, 2c6o, 4kmn, 1m17, and 4wt2). On the other molecular side, compounds **7b** and **7c** enhanced the relative expression of P53 and Bax (apoptotic genes). On the other hand, they downregulated the anti-apoptotic genes Bcl2 and CDK4. The activity of caspase-3, caspase-8, and caspase-9 was significantly increased by the studied compounds. So, the results suggested that compounds **7b** and **7c** induced the extrinsic and intrinsic pathways of apoptosis in A549 cells. The flowcytometric analysis demonstrated that compound **7b** arrested the cell cycle at G0/G1 and S phases and induced apoptosis and necrosis by a percentage of 24.93% and 17.23% respectively. The SAR revealed that compound **7b**with thiophene moiety was more active than compound **7c** with pyridine ring. At the end of our studies, the authors proposed that both theoretical and experimental studies completed each other and confirmed the enhanced and effective role of novel two chalcones **7b** and **7c** on apoptosis of lung cancer cell line (A549). The two chalcones exerted both intrinsic and extrinsic pathways of apoptosis to die cancer cells and prevent its growth.

## Supplementary Information

Below is the link to the electronic supplementary material.

Supplementary file 1 (DOCX 3879 KB)

## Data Availability

Data available on request.
