# Supplementary material for: Theoretical and molecular mechanistic investigations of novel (3-(furan-2-yl)pyrazol-4-yl) chalcones against lung carcinoma cell line (A549)
Source: Naunyn Schmiedebergs Arch Pharmacol. 2022 Dec 5;396(4):719–36. doi: 10.1007/s00210-022-02344-x (PMC10042774; doi:10.1007/s00210-022-02344-x)
Supplement: Supplementary file 1 — (DOCX 3879 KB) [file 210_2022_2344_MOESM1_ESM.docx]

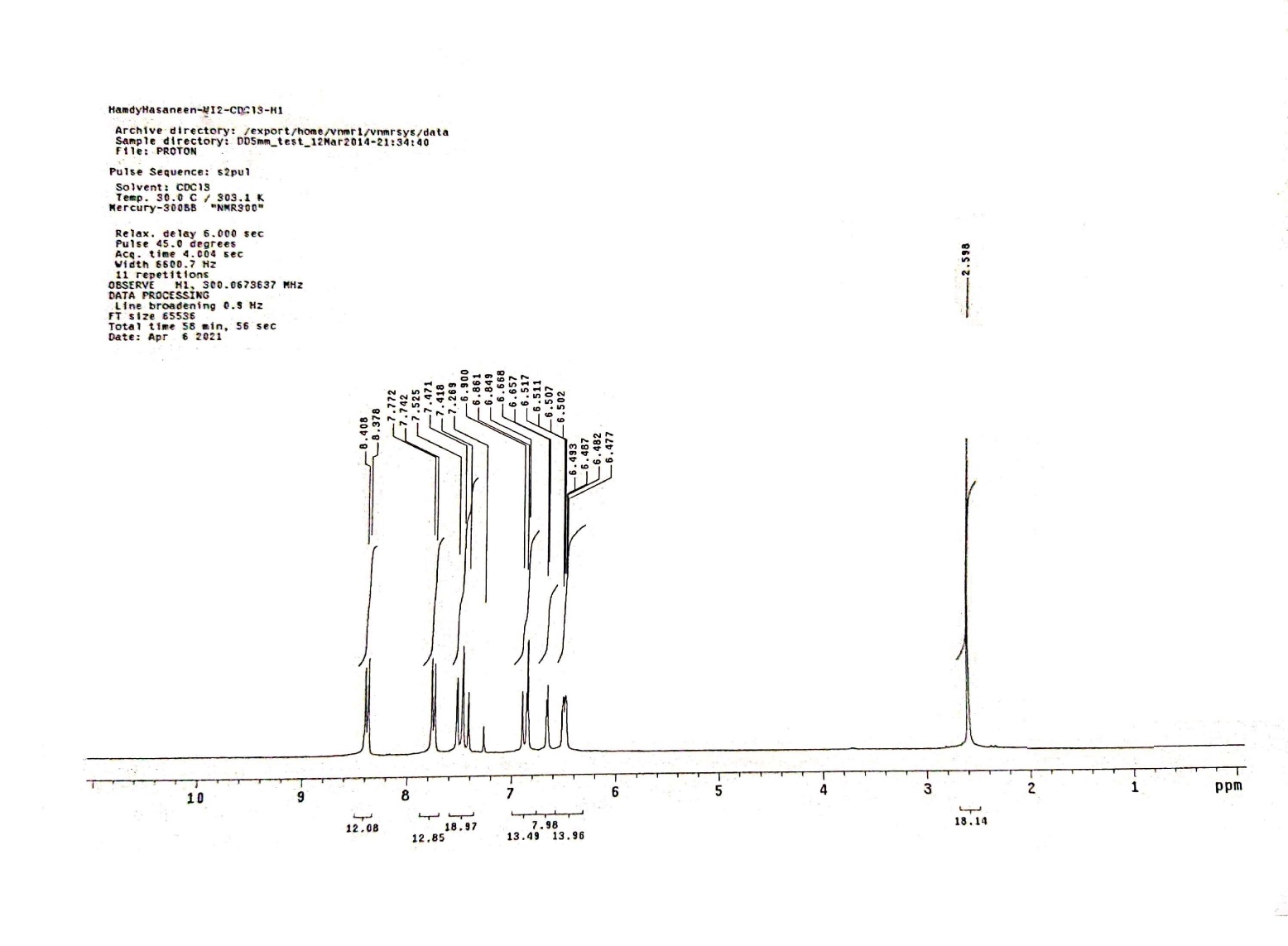

**^1^H NMR of Compound 7a**


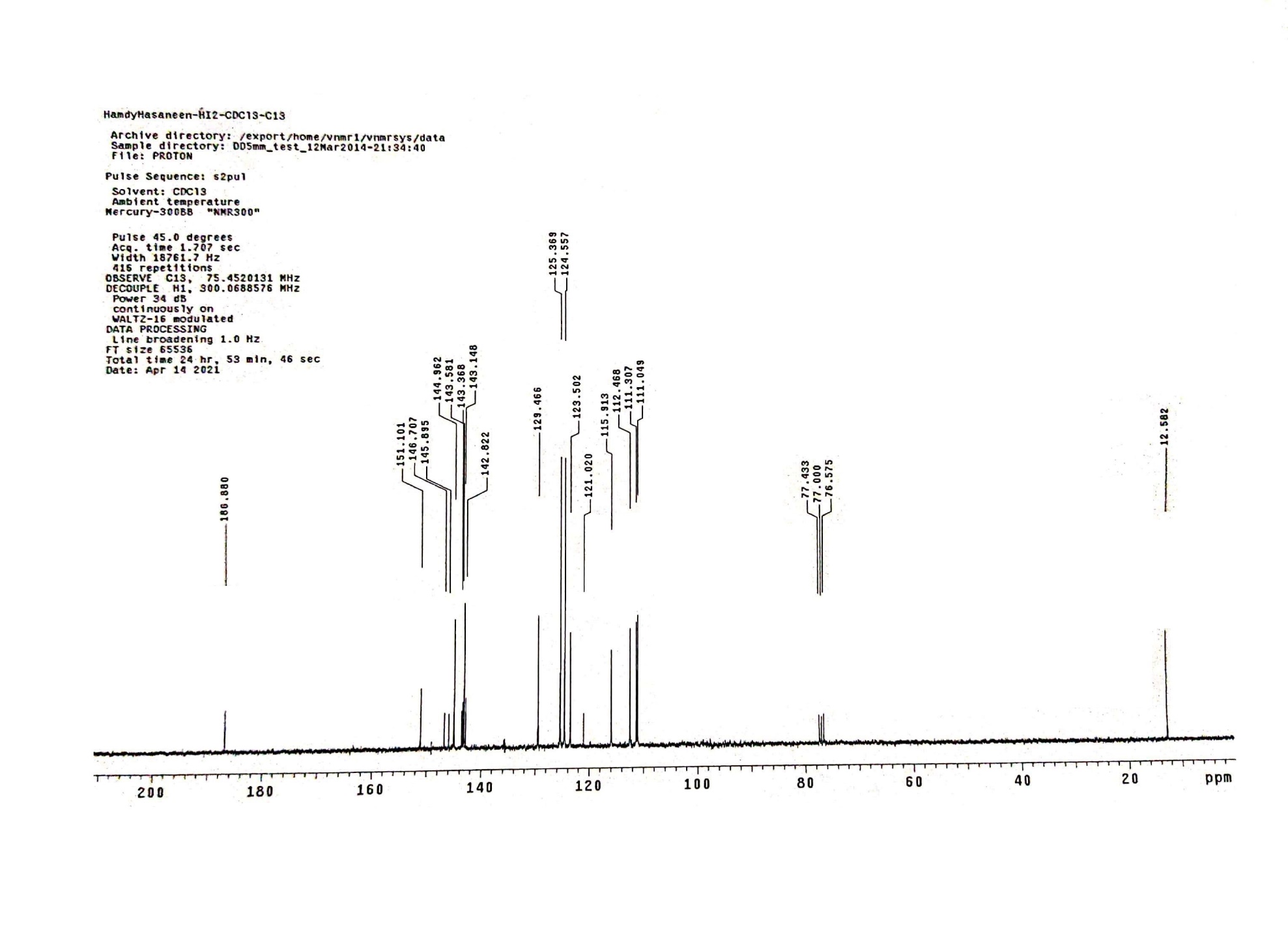

**^13^C NMR of Compound 7a**


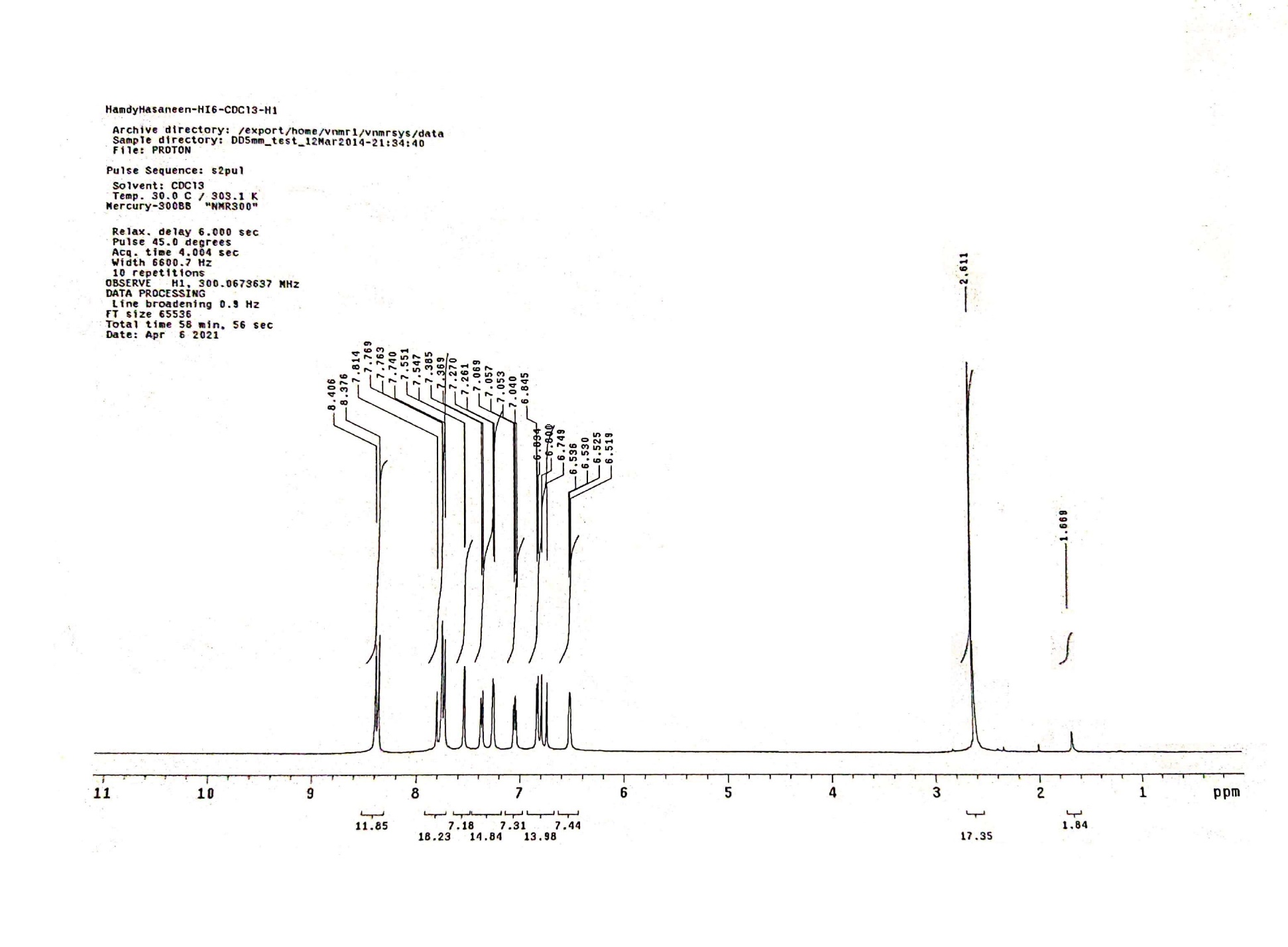

**^1^H NMR of Compound 7b**


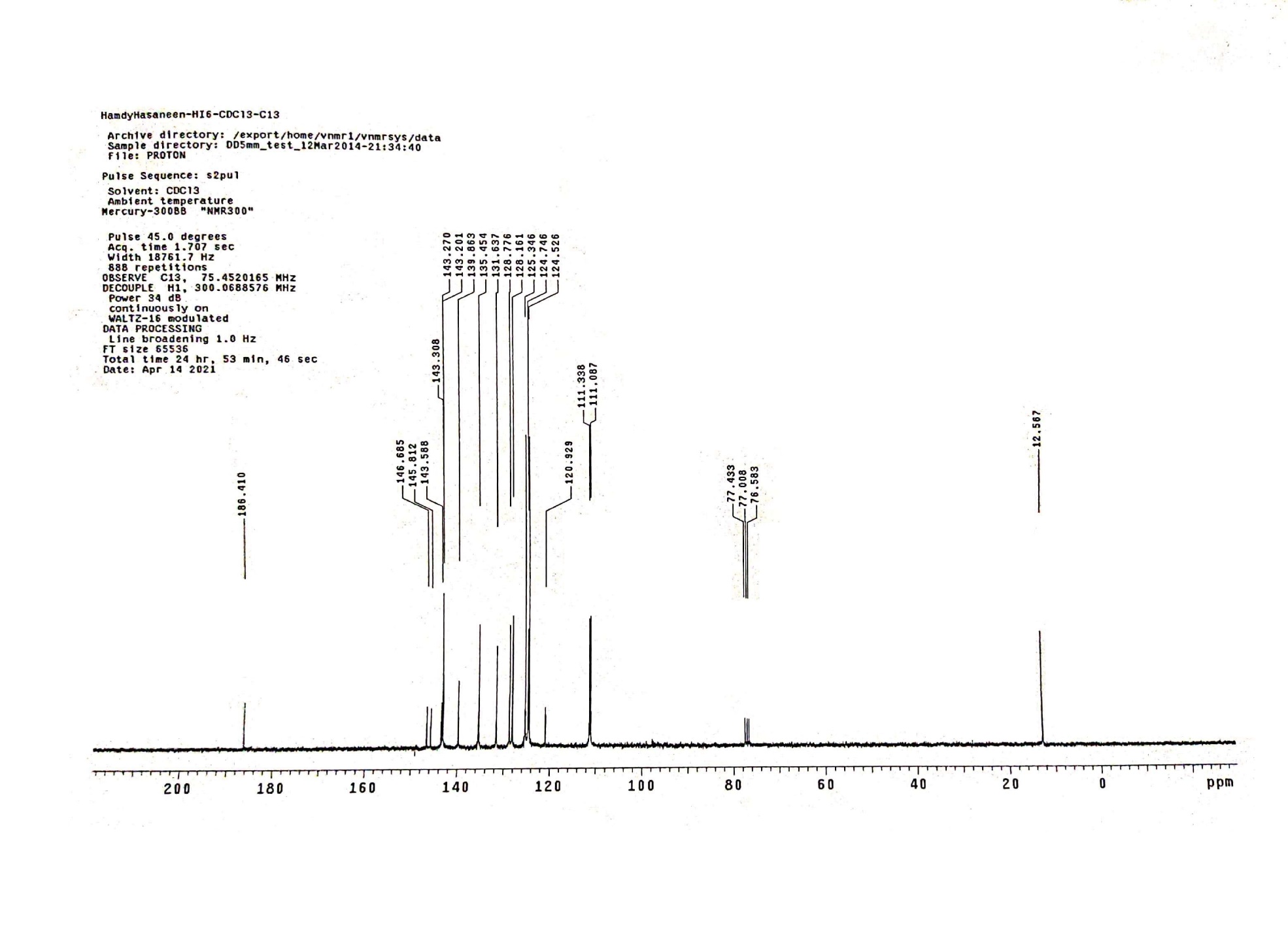

**^13^C NMR of Compound 7b**


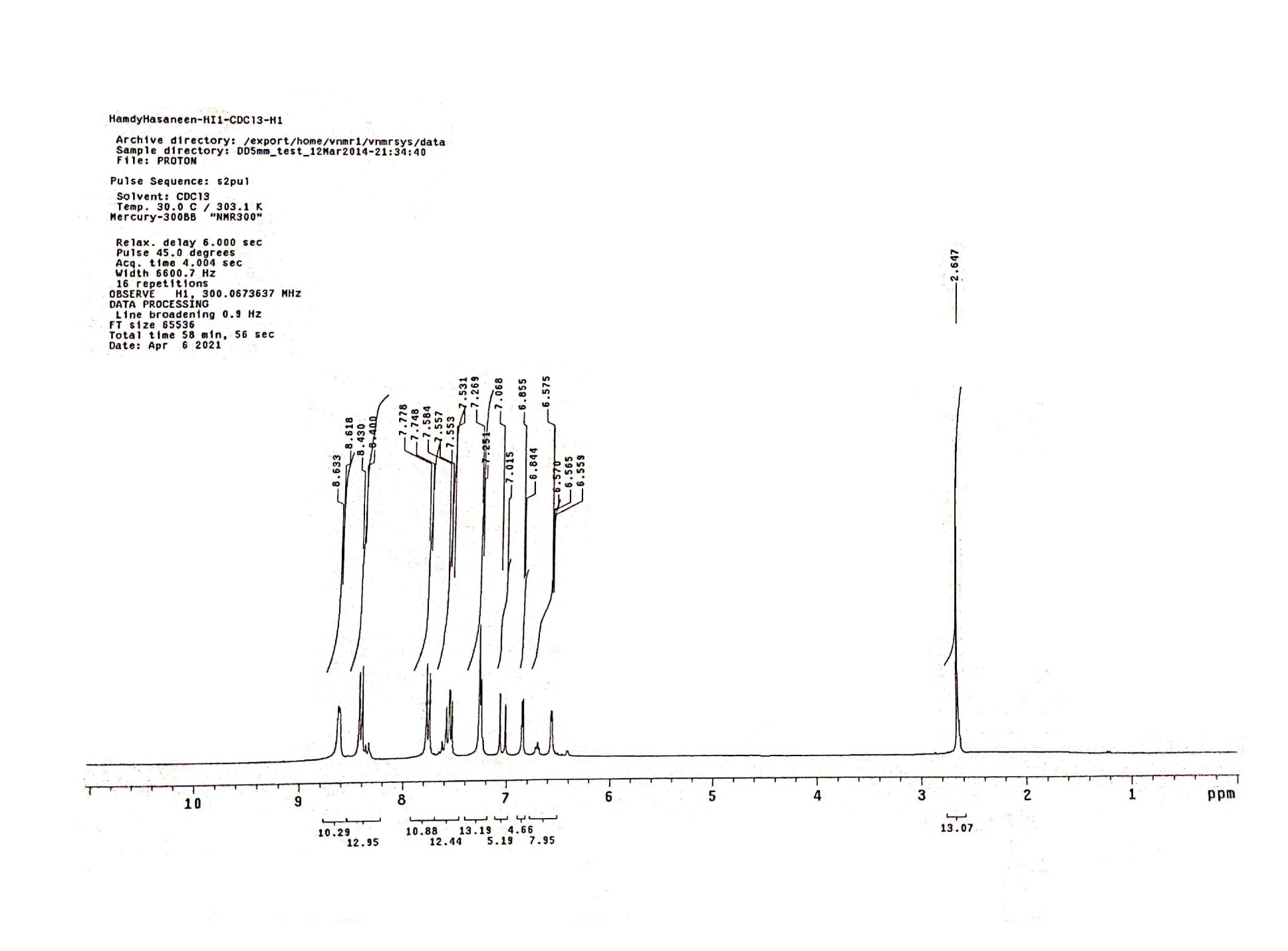

**^1^H NMR of Compound 7c**


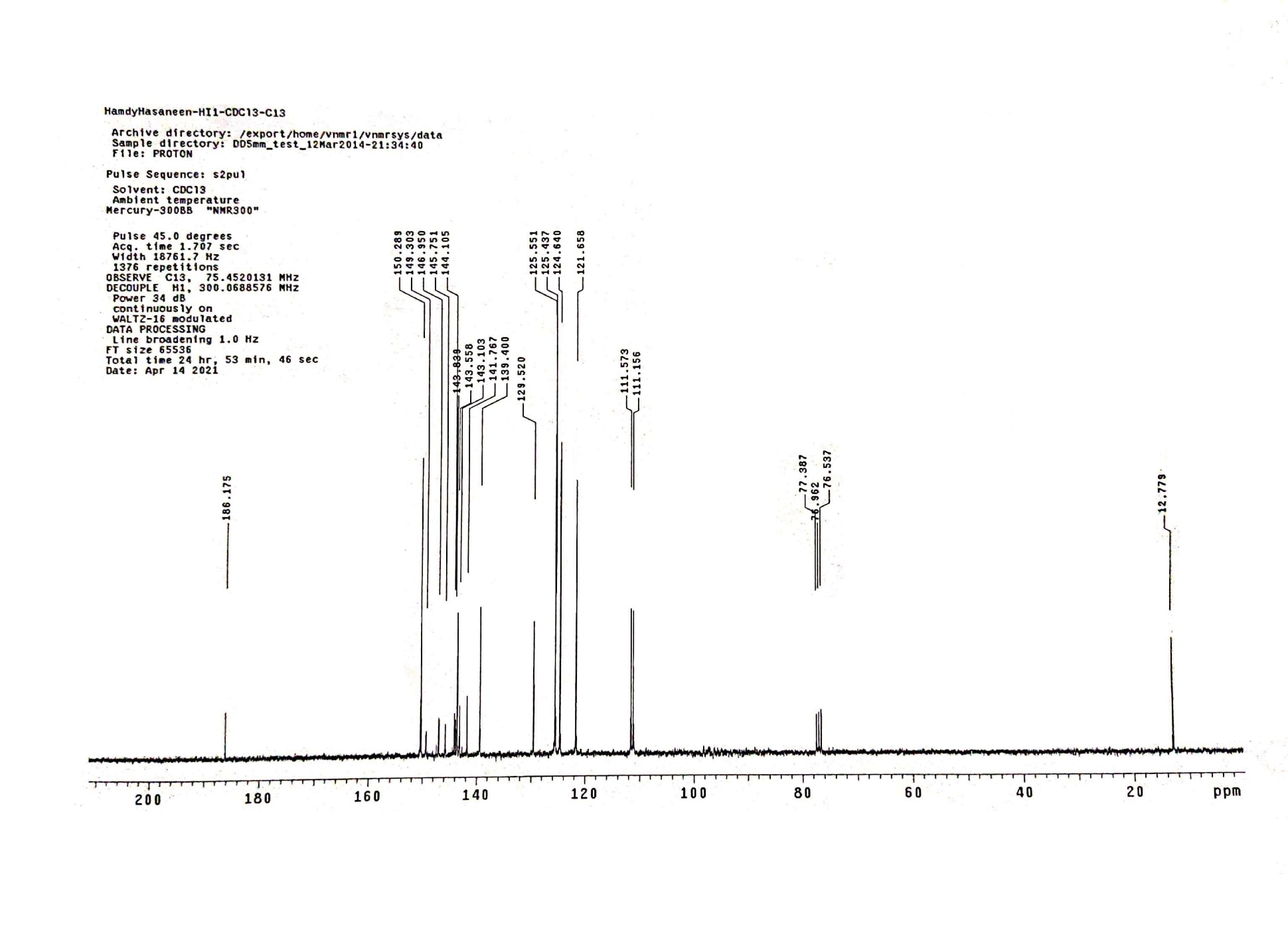

**^13^C NMR of Compound 7c**


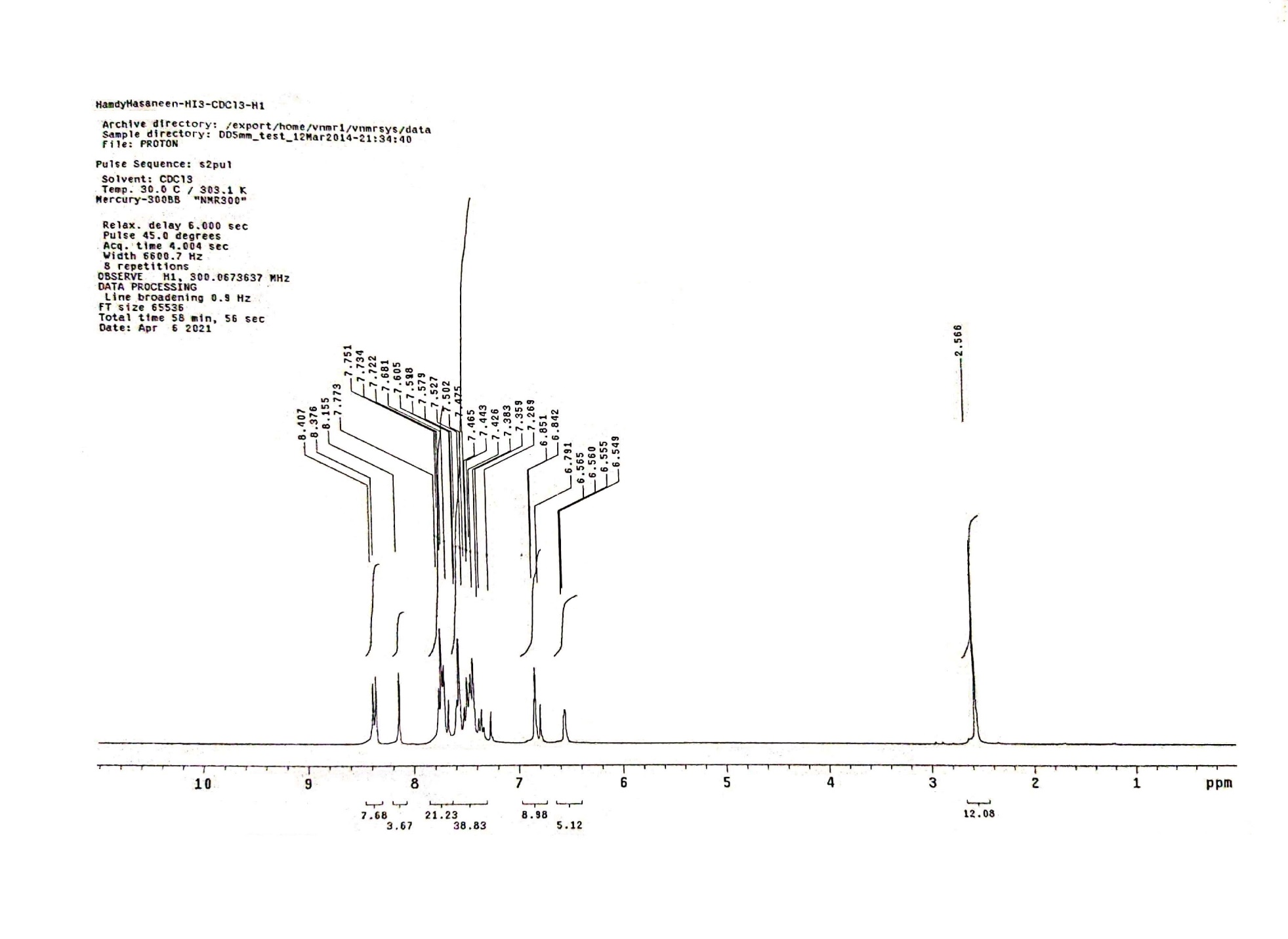

**^1^H NMR of Compound 7d**


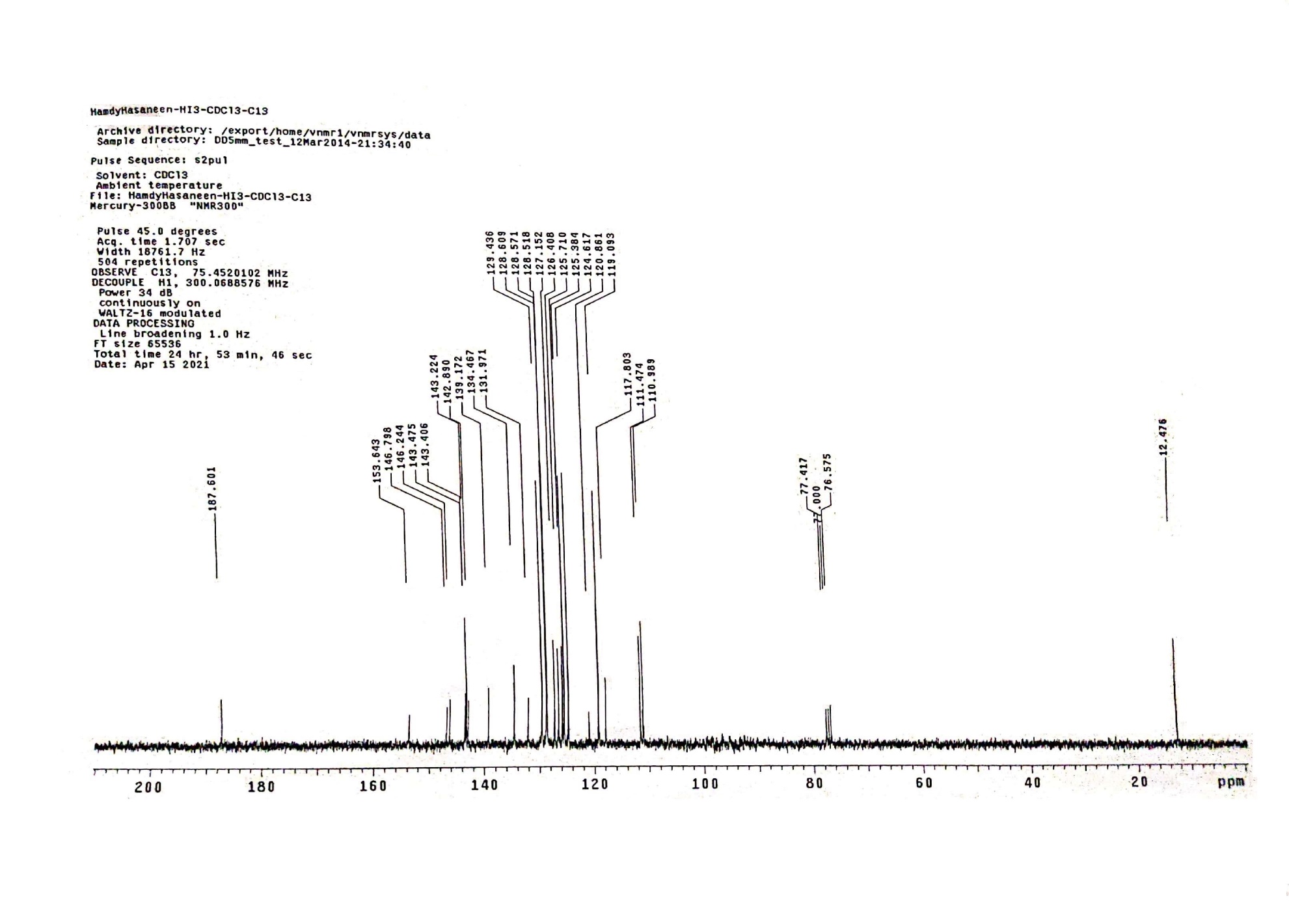

**^13^C NMR of Compound 7d**


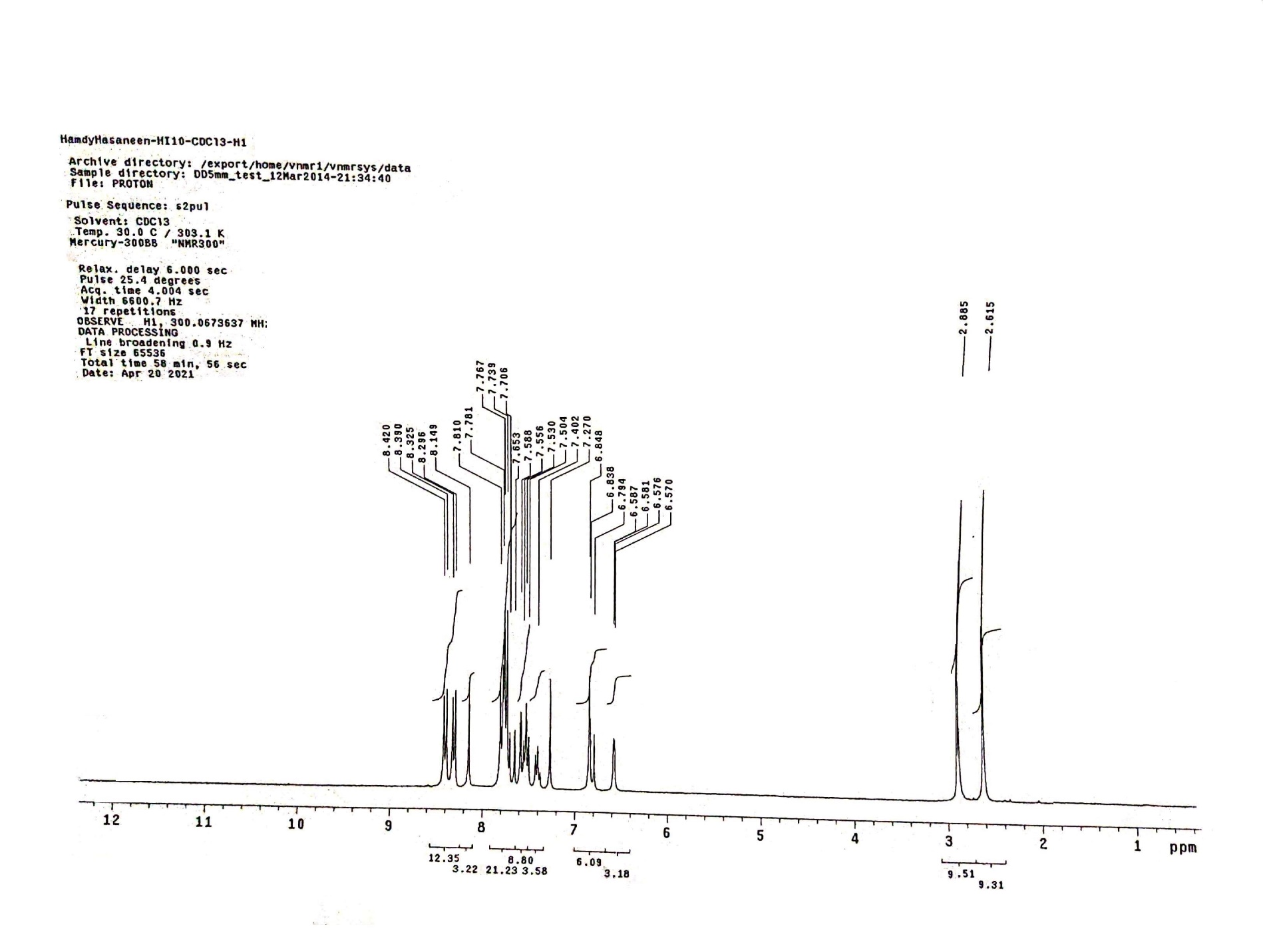

**^1^H NMR of Compound 7e**


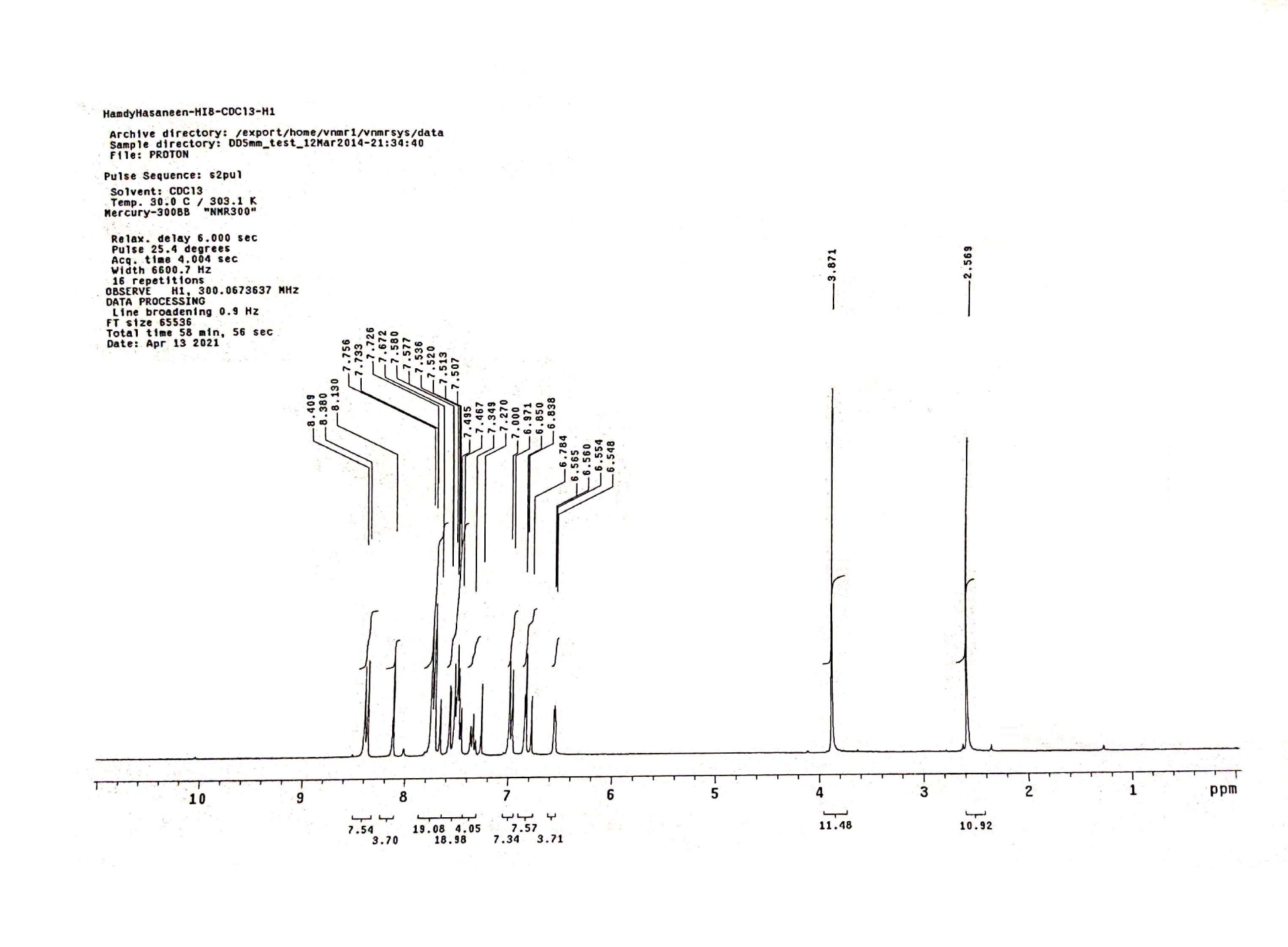

**^1^H NMR of Compound 7f**


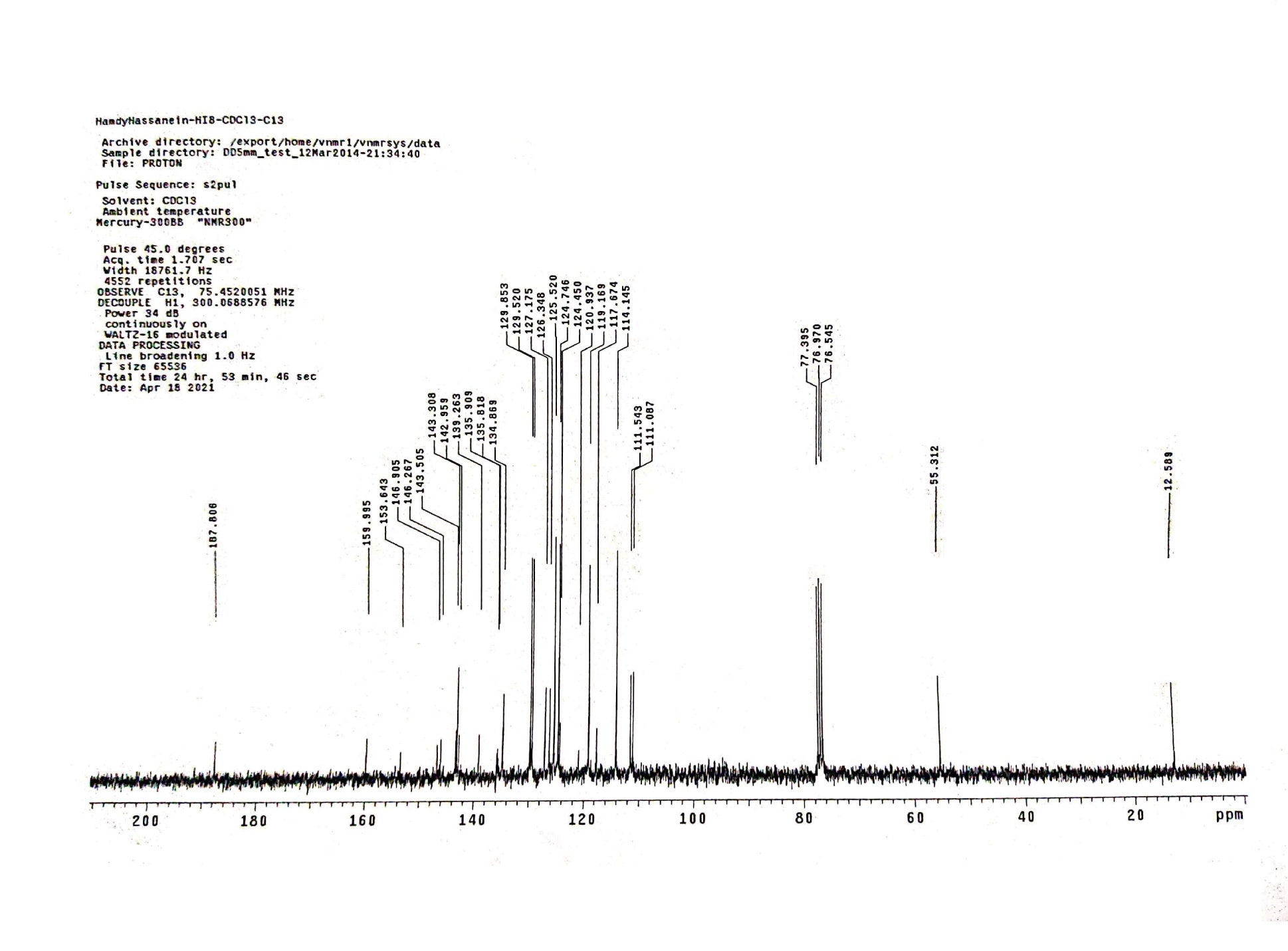

**^13^C NMR of Compound 7f**


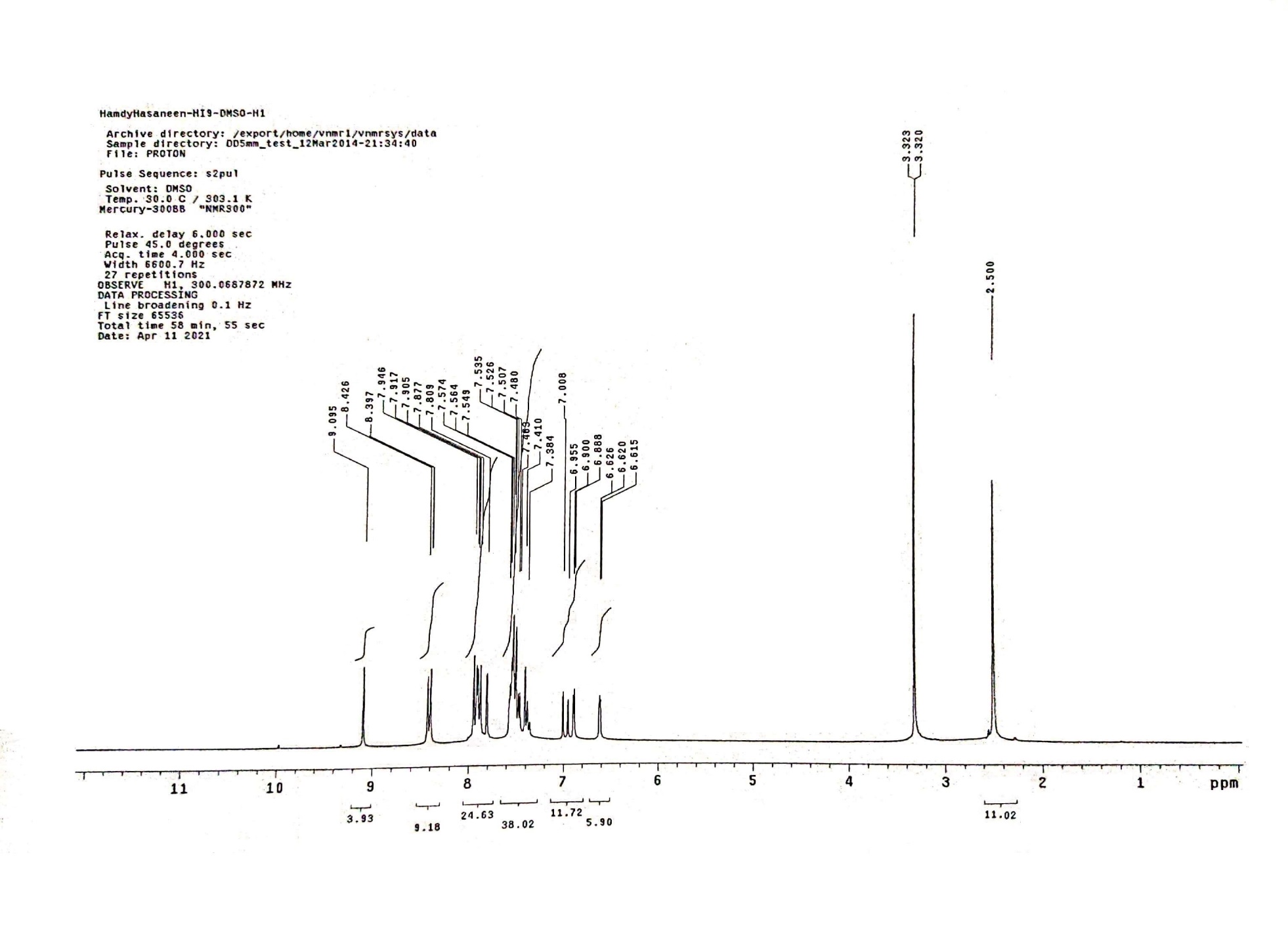

**^1^H NMR of Compound 7g**


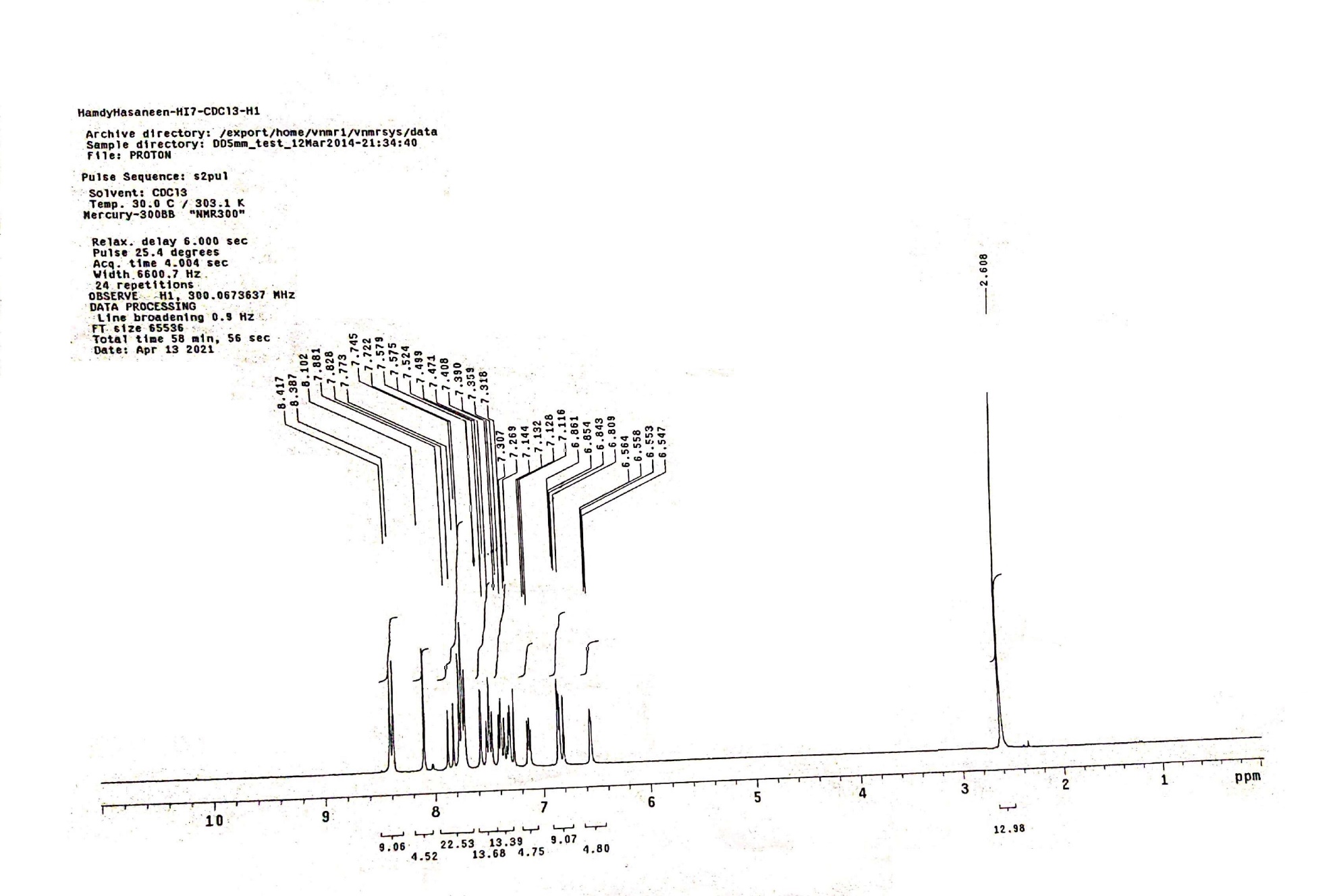

**^1^H NMR of Compound 7h**


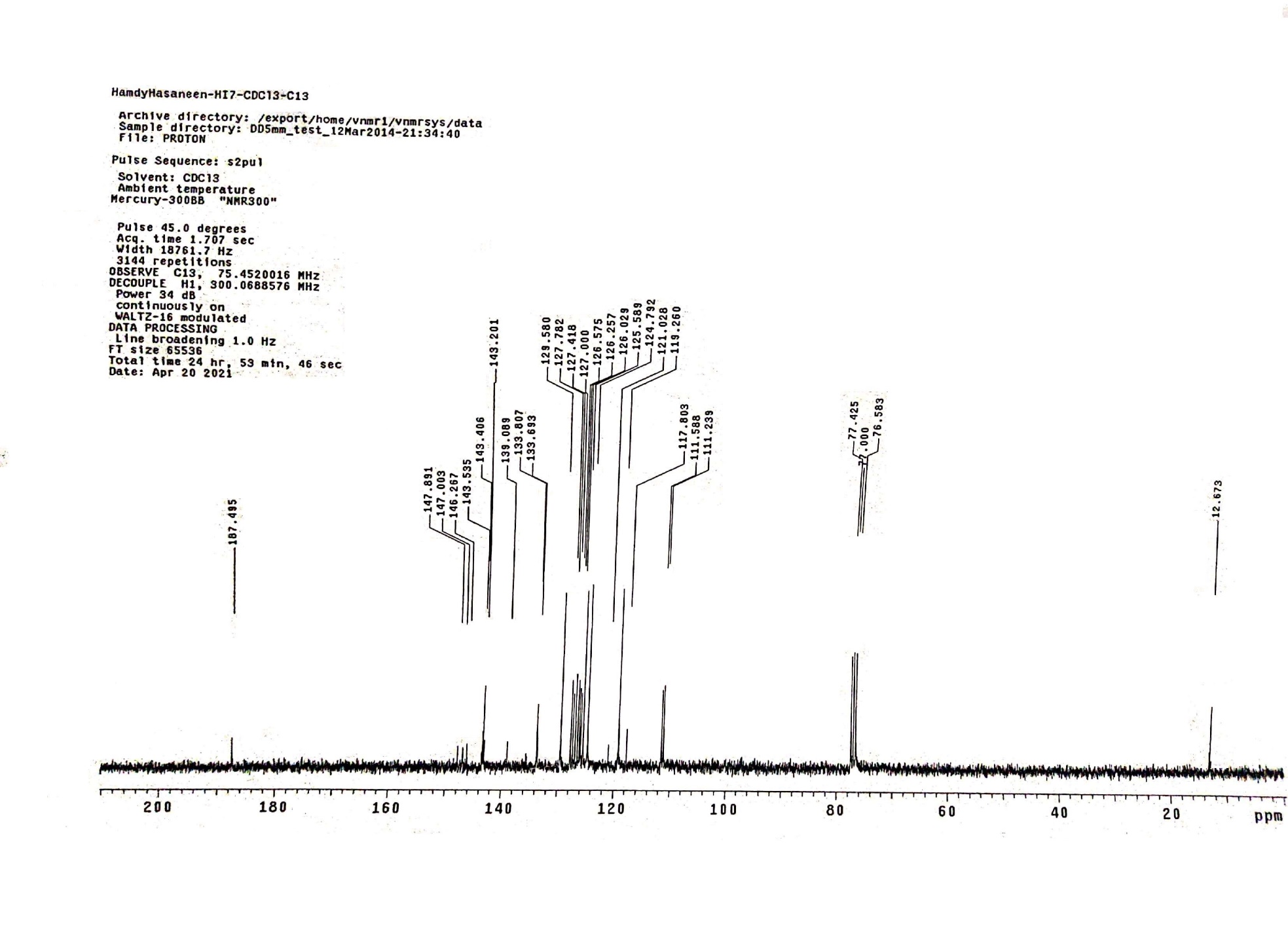

**^13^C NMR of Compound 7h**
